# Development of a novel anti-CEACAM5 VHH for SPECT imaging and potential cancer therapy applications

**DOI:** 10.1007/s00259-025-07321-z

**Published:** 2025-05-13

**Authors:** Ying Cong, Rianne Biemans, Natasja G. Lieuwes, Dennis Suijlen, Philippe Lambin, Ingrid Dijkgraaf, Matthias Bauwens, Ala Yaromina, Ludwig J. Dubois

**Affiliations:** 1 The M-Lab, Department of Precision Medicine, GROW– Research Institute for Oncology and Reproduction, Maastricht University, UNS50/23, Maastricht, PO Box 616, 6229 ER Maastricht, The Netherlands; 2 Department of Biochemistry, Cardiovascular Research Institute Maastricht (CARIM), Maastricht University, Maastricht, The Netherlands; 3 Department of Radiology and Nuclear Medicine, Maastricht University Medical Center, Maastricht, The Netherlands

**Keywords:** CEACAM5, ^99m^Tc-VHH, SPECT imaging, Biodistribution, VHH-based drug conjugate

## Abstract

**Purpose:**

In this study, we investigated the utility of a novel developed anti-CEACAM5 VHH for cancer diagnosis and its potential of being a targeting-moiety of VHH-drug conjugates for cancer therapy.

**Methods:**

Anti-CEACAM5 VHH (6B11) affinity and specific cellular binding was confirmed by ELISA, FACS and immunofluorescence in cancer cell lines with varying CEACAM5 expression levels. Intracellular penetration ability within tumor spheroids was tested with Oregon Green 488 labeled 6B11 (OG488-6B11). Biodistribution and binding specificity of ^99m^Tc-radiolabeled 6B11 was tested in A549 CEACAM5 overexpressing (A549-CEA5-OV) and knockout (A549-CEA5-KO) tumor-bearing mice upon SPECT/CT imaging, γ-counting and autoradiography. The therapeutic efficacy of 6B11 and 6F8 (anti-CEACAM5 VHH with lower binding affinity) was tested by viability, wound healing and adhesion assays. To verify the potential of VHHs as a warhead for VHH-drug conjugation, an internalization assay with OG488 labeled VHH was performed.

**Result:**

6B11 demonstrated high binding affinity (EC_50_ 0.5nM) and cellular binding. OG488-6B11 penetrated tumor spheroids completely at 24 h, while a conventional antibody was only visible at the spheroid periphery. SPECT imaging indicated higher uptake (*p* < 0.05) in A549-CEA5-OV tumors, resulting in increased tumor-to-blood ratios especially at 4 (2.0016 ± 1.1893, *p* = 0.035) and 24 (2.9371 ± 2.0683, *p* = 0.003) hpi compared to A549-CEA5-KO tumors at 4 (0.5640 ± 0.3576) and 24 (0.8051 ± 0.4351) hpi. ^99m^Tc-6B11 was predominantly renally cleared. Autoradiography and immunohistochemistry confirmed these uptake patterns. 6B11 nor 6F8 did exhibit significant anti-cancer therapeutic efficacy in vitro. OG488-6B11 was effectively internalized and accumulated in cells in a time-dependent manner, to end up in the lysosomes.

**Conclusion:**

The anti-CEACAM5 VHH 6B11 is a good candidate for SPECT-based cancer diagnosis and can be potentially used as targeting moiety in the development of VHH-based drug conjugates for cancer treatments.

**Graphical Abstract:**

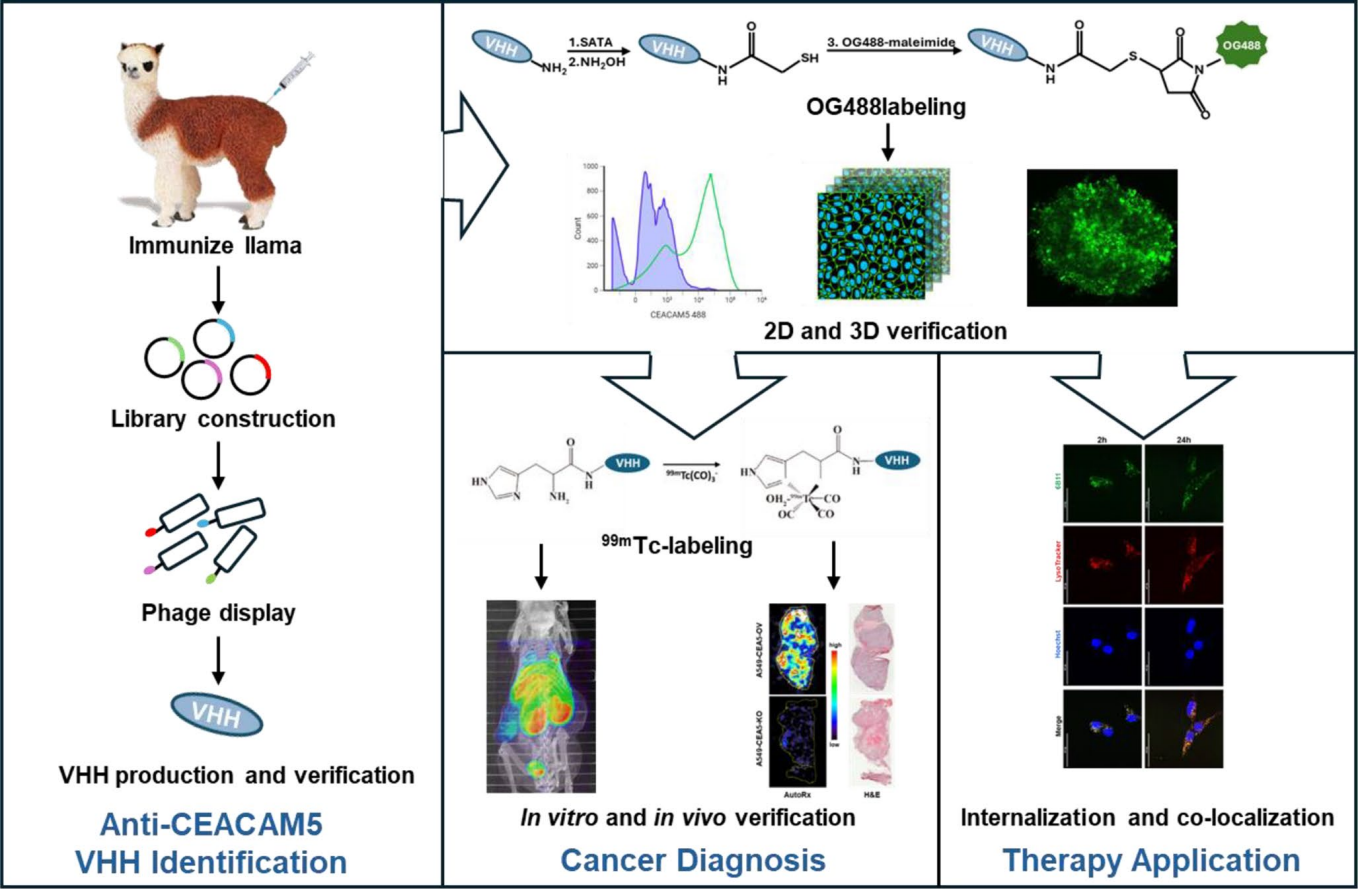

**Supplementary Information:**

The online version contains supplementary material available at 10.1007/s00259-025-07321-z.

## Introduction

Non-invasive molecular imaging using imaging agents, containing a targeting moiety and a radioisotope or fluorescent moiety, is one of the important tools in cancer research for diagnosis as well as for monitoring cancer progression and therapy effectiveness [1]. Although ^18^F-FDG is considered the golden standard for tumor imaging, in clinical practice it does not provide detailed information about e.g. target expression patterns in context of targeted anti-cancer therapies. Therefore, more specific biomarker-based tracers are needed. Conventional antibodies have been used increasingly in imaging studies based on their high specificity and sensitivity accompanied with low toxicity [2]. Their large molecular size and relative slow blood clearance, however, requires the use of radionuclides with a long physical half-life resulting in more radiation exposure and additional measures for radiation protection [3]. The variable domain of heavy chain of heavy-chain only antibodies (VHHs) are the smallest antibody-based fragments that can recognize antigens specifically [4]. VHHs show great potential as nuclear imaging agents due to their small molecular weight, deeper penetration into the central part of solid tumors and fast blood clearance, leading to high tumor-to-background ratios at earlier time points after injection [5–7]. Currently, there are multiple VHH-based radioactive probes in preclinical development and in clinical trials [8–10]. VHHs show also promise for the development of therapeutic agents, such as antibody-drug conjugates (ADCs) [11]. The high targeting properties of antibodies combined with the high efficacy of the small molecule drug make ADC an emerging targeted therapy with great potential in the field of cancer treatment. The antibody component should ideally have low immunogenicity, high target specificity, high binding affinity, good stability and the ability to lead to efficient endocytosis [12]. In the last years, many studies have tried to use other forms of antibodies, such as bispecific antibodies and VHHs, to further improve the safety and efficacy of ADCs [13–16].

The Carcinoembryonic Antigen-Related Cell Adhesion Molecule 5 (CEACAM5) is a highly glycosylated protein, first recognized by Gold and Freeman in colon cancer in 1965 [17]. In cancer tissue, CEACAM5 is highly expressed on the membrane of cells in epithelial cancer, including colon cancer, stomach cancer, lung cancer, pancreatic cancer, cervical cancer and others [18, 19]. The extracellular domain of CEACAM5 is responsible for cell adhesion, while the intracellular domain for intracellular signaling pathway activation. As a cell adhesion molecule, in order to maintain cellular communication and tissue structure, CEACAM5, via its N domain, facilitates cell-cell and cell-matrix interaction through both homophilic (CEACAM5 to CEACAM5) and heterophilic (CEACAM5 to non-CEACAM5 molecule) binding [20]. Additionally, CEACAM5 participates in regulating cell proliferation and differentiation [21]. CEACAM5 overexpression inhibits anoikis, leading to the disruption of tissue architecture and therefore promoting tumor growth and metastasis formation [18, 22], exemplified by its overexpression in lung metastases derived from breast cancer [23]. Soluble CEACAM5, i.e. release of its extracellular segment upon degradation by phosphatidylinositol-specific phospholipase C/D (PI-PLC/D), induces proangiogenic effects through activation of endothelial cells via integrin β-3, focal–adhesion kinase and s-Src kinase and their downstream MAP-ERK and PI3K/Akt pathways [24]. As CEACAM5 is highly expressed on the membrane of cells in a variety of epithelial cancers [18, 19, 25], it has been investigated as a radiolabeled biomarkers for future clinical cancer diagnosis [26]. Imberti et al. labeled labetuzumab, an anti-CEACAM5 monoclonal antibody, with ^89^Zr and demonstrated accumulation in neuroendocrine prostate cancer H660 xenografts [27]. Wang et al. generated a ^99m^Tc-labeled anti-CEACAM5 VHH, which rapidly accumulated in lung cancer H460 xenografts in vivo assessed by γ-counting, but did not show target specificity [28].

In this study, we developed novel anti-CEACAM5 VHHs with good binding affinity and specificity with the aim of testing their diagnostic and therapeutic potential. Furthermore, we investigated the cellular distribution and penetration as well as internalization capability to demonstrate the potential for VHH-drug conjugation for therapeutic application.

## Materials and methods

Extended information, protocols and experimental procedures are provided in supplementary materials.

### Anti-CEACAM5 VHH production, ^99m^Tc and OG488 conjugation

The selected VHHs were produced in E. coli BL21 (DE3) followed by purification using HisPur™ Ni-NTA Spin Purification Kit (Thermofisher, 88229) and Spin Desalting Columns (Thermofisher, 89894) or Pierce™ Protein Concentrators (Thermofisher, 88526). VHH yield was determined using Pierce™ BCA Protein Assay Kits (Thermofisher, A55864). The purity of produced VHHs were verified by Coomassie staining.

For labeling VHH with ^99m^Tc, IsoLink kit for tricarbonyl (Center for Radiopharmaceutical Sciences) was used and the labeling was performed according to kit’s instructions. The ^99m^Tc-labeled product was tested by TLC/HPLC. OG488 was labeled with lysine residues via a SATA reaction. The OG488-labeled product was tested by MALDI-TOF-MS.

### Cell culture

All cells were cultured routinely in a 5% CO_2_ incubator at 37 °C. Lung cancer A549 (ATCC) and A549 CEACAM5 knockout (A549-CEA5-KO, Abcam, ab287319) cells were cultured in DMEM (Sigma-Aldrich) with 10% (v/v) FBS (Sigma-Aldrich). A549 CEACAM5 overexpressing cells (A549-CEA5-OV, kind gift from Dr. Bernhard Singer) were cultured in DMEM with 10% (v/v) FBS and 1 mg/mL Geneticin (G418) [21]. Lung cancer H292 cells were cultured in RPMI1640 (Sigma-Aldrich) with 10% (v/v) FBS.

### Spheroid formation and VHH diffusion assay

Single cells were seeded in an ultra-low attachment plate (7007, Corning) and plates were incubated at 37 °C / 5% CO_2_ for minimally 3 days to enable spheroid formation. Upon reaching a diameter of approximately 500 μm, spheroids were incubated at 37 °C / 5% CO_2_ for 1, 3 and 24 h with culture medium containing 25 nM of OG488-6B11 or alexa488-anti-CEACAM5 antibody [EPCEAR7] (ab214868, Abcam). Images were taken with 40× magnification using a Leica SPE confocal microscope with z-stack mode. For the method of analyzing results please refer to the description in Supplementary Material and Methods.

### In vivo study to test ^99m^Tc-VHH binding specificity and biodistribution in tumor-bearing mice

All animal experiments were in accordance with local institutional guidelines for animal welfare and approved by the Animal Ethical Committee of the University of Maastricht (AVD10700202216527). Mycoplasma-free A549-CEA5-KO cells (1.5 × 10^6^ cells in 50 µL matrigel, Corning, 354234) were injected subcutaneously in the right flank of eight-week-old female Crl:NU(NCr)-Foxn1^nu^ mice (Charles River). When palpable tumors were formed, A549-CEA5-OV cells (1.5 × 10^6^ cells in 50 µL matrigel) were injected subcutaneously in the contralateral flank. When tumor volume reached 623 ± 339 mm^3^, mice were intravenously injected with ^99m^Tc-6B11 (64.9 ± 21.5 MBq representing 4.99 ± 1.65 nmoles) under isoflurane inhalation anesthesia (4% induction, 2.5% maintenance). microSPECT (U-SPECT, MILabs B.V.) image acquisition was performed under isoflurane anesthesia at 1, 4 and 24 h post tracer injection. After each microSPECT acquisition, a high-resolution microCT image was acquired using the X-RAD 225Cx (Precision X-Ray Irradiation Inc.), while the animal remained positioned in a clickable bed. After the last imaging session, mice were killed by cervical dislocation. Tissue biodistribution of ^99m^Tc-6B11 as well as histological analysis of formalin fixed tumors were performed as described in supplementary Materials and Methods.

### Internalization assay

Cells were seeded in 35 mm confocal dishes (81156, ibidi) at a density of 5 × 10^4^/dish. The next day, growth medium was removed, and cells were washed with pre-warmed serum-free culture medium and incubated with serum-free medium at 37 °C for 1 h. To stop endocytosis, cells were placed at 4°C for 15 min. Afterwards, cells were incubated with 2 µg/mL OG488-6B11 (4°C for 30 min), washed twice with pre-cooled serum-free medium and then incubated at 37 °C for different time points (0, 1, 2, 4, 24 and 48 h). For lysosome labeling, 50 nM LysoTracker (L12492, Invitrogen) was added to the cells 2 h prior to the following washing step. After each incubation time, cells were washed twice with pre-cooled PBS and then incubated with pre-cooled high-salt-low-pH (HSLP) buffer (4°C for 2 min), followed by washing twice with pre-cooled PBS. Cells were fixed with 4% PFA (4°C for 15 min), washed with PBS and incubated with the Hoechst 33342 nucleic acid stain (1 µg/mL) at RT for 5 min. Then, cells were washed twice with PBS and immersed in mounting buffer. Images were taken with a Leica SPE confocal microscope at 630× magnification and colors from different channels were merged using FIJI.

### Statistics

All data processing and statistical analysis were performed with GraphPad Prism v10 software; data are presented as the mean ± standard deviation. P values were determined using two-way ANOVA or Mann-Whitney U tests, and p values less than 0.05 indicated statistically significant difference.

## Results

### VHH generation, production and validation

**Fig. 1:**
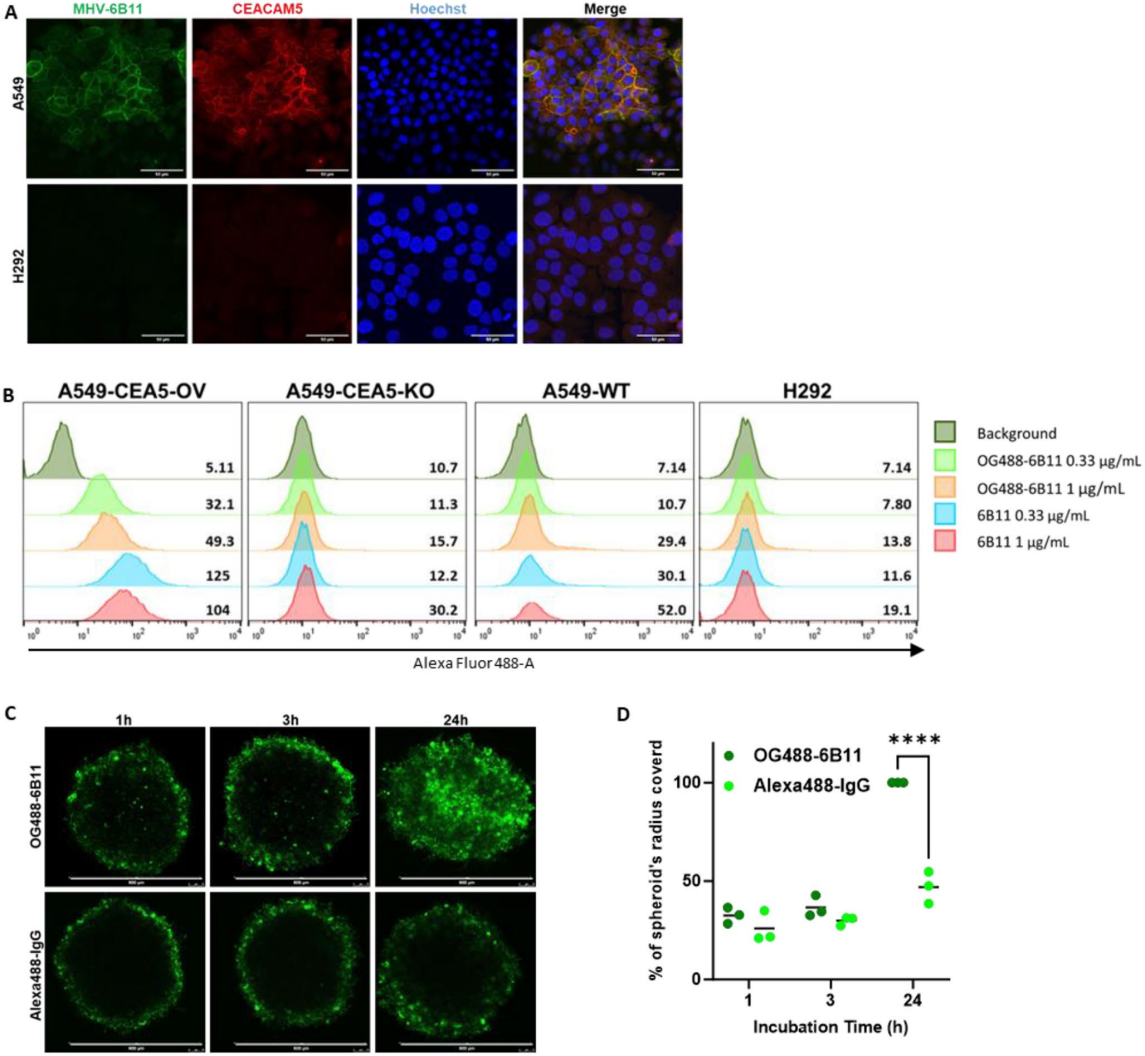
Cellular binding and distribution of fluorescent labeled VHH. (**A**) Co-localization of 6B11 (green) with anti-CEACAM5 commercial antibody (red) in A549 and H292 cells. Cell nuclei (Hoechst 33342) is shown in blue. Scale bar = 50 μm. (**B**) Cellular binding of OG488-6B11 and 6B11 at different VHH concentrations in CEACAM5 overexpressing A549 (A549-CEA5-OV), CEACAM5 knockout A549 (A549-CEA5-KO), wild type A549 cells and in CEACAM5 negative H292 cells tested by flow cytometry. (**C**) Diffusion of Alexa488-IgG and OG488-6B11 in a representative A549-CEA5-OV spheroid after 1, 3 and 24 h incubation. Scale bar = 600 μm. (**D**) The displacement profile of Alexa488-IgG and OG488-6B11 VHH in A549-CEA5-OV spheroids after different incubation times. Lines indicate the mean value of *n* = 3. **** *p* < 0.0001. Of note: data for 6B11 as control are also used in Fig. 2C

Human CEACAM5 was used for immunization of llamas to construct the library and 11 novel anti-CEACAM5 VHHs were selected from phage display (Fig. S1). To test VHH specificity, cancer cell lines with low (H292) and high CEACAM5 expression (A549), as confirmed with western blotting and immunocytochemistry, were selected (Fig. S2A, S2C). In addition, A549 cells overexpressing CEACAM5 (A549-CEA5-OV) and A549 CEACAM5 knockout (A54-CEA5-KO) cells were used (Fig. S2B). Amongst 11 VHHs tested, 6B11 and 6F8 were selected as lead candidates based on their superior apparent affinity (0.30 nM and 1 nM respectively, Fig. S1I) and high co-localization with a commercial antibody-based CEACAM5 membranous staining (Fig. S3) assessed by the DICE coefficient of similarity (Fig. S2D). An in-house developed ELISA confirmed the high binding affinity of 6B11 (0.5 nM) and 6F8 (5.05 nM) VHHs amongst 6 potential candidates tested (Fig. S2E). Moreover, the mean fluorescence intensity of the 6B11 and 6F8 VHHs bound to the A549-CEA5-OV cells increased with increasing concentration of the VHH in the medium (Fig. S2F). In an independent experiment, ICC confirmed co-localization of CEACAM5 with 6B11 VHH in A549, while lack of staining in H292 (Fig. 1A). To produce sufficient amount of VHH for successive experiments, an E. Coli VHH expression system was constructed and VHH production and purification methods were established (Figure. S4, S5A) [29]. In house produced 6B11 VHH confirmed to have higher apparent affinity (1.21 nM) as compared to 6F8 (5.9 nM, Fig. S5B). Based on these results, 6B11 VHH was selected as lead VHH for the subsequent experiments. Further characterization of 6B11 showed a dose dependent binding of 6B11 in A549-CEA5-OV, but not in A549-CEA5-KO cells (Fig. S5C). MALDI-TOF-TOF-MS of 6B11 confirmed a measured molecular weight of 14.23 kDa (Fig. 5SD).

To further confirm the binding specificity and penetrating ability of 6B11 in a 3D cellular model, VHH was conjugated with Oregon Green 488 (OG488). The resulting product, OG488-6B11, was a mixture of 6B11 tagged with a 1×, 2× or 3× OG488 moiety as determined by MALDI-TOF-TOF-MS, with an average molecular weight of 15 kD (Fig. S6). Cellular binding affinity of OG488-6B11 was dependent on the dose and target expression levels, but overall, 7-fold lower compared to 6B11 (Fig. 1B, S7). The cellular diffusion characteristics of OG488-6B11 were compared with an alexa488 labeled anti-CEACAM5 conventional antibody in A549-CEA5-OV spheroids. OG488-6B11 penetrated A549-CEA5-OV spheroids completely within 24 h incubation, while the conventional antibody remained in the periphery (Fig. 1C and D). No staining was observed in CEACAM5-negative A549-CEA5-KO and H292 spheroids supporting binding specificity of OG488-6B11 (Fig. S8).

### ^99 m^Tc-VHH labeling and in vitro binding characteristics

**Fig. 2:**
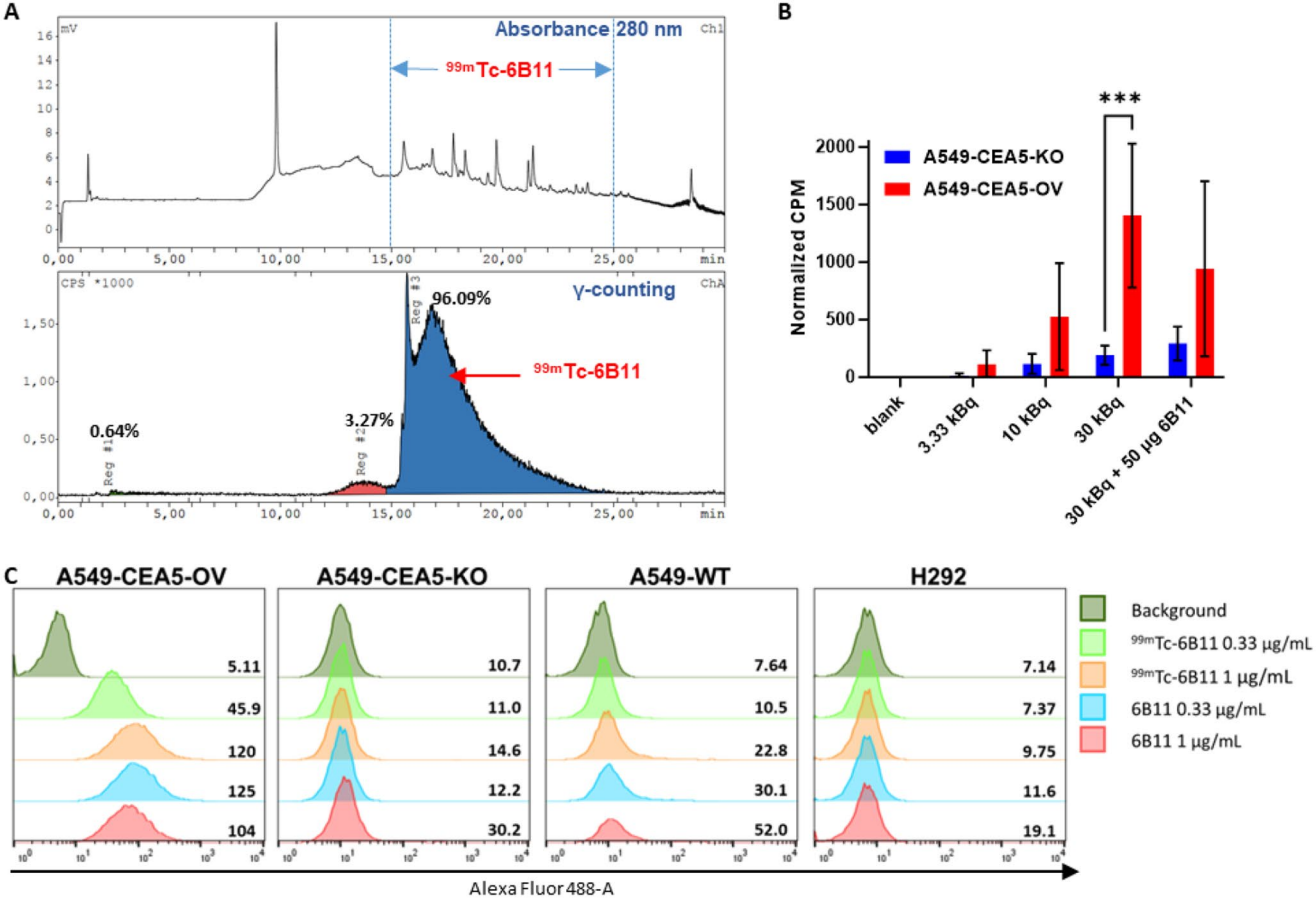
Radiolabeling and in vitro validation of ^99m^Tc-6B11. (**A**) HPLC result of purified ^99m^Tc labeled anti-CEACAM5 VHH 6B11 using 280 nm absorbance and γ counting assays. The percentage of peak area is indicated next to the peak. The peak between retention time 15 ~ 25 min indicated ^99m^Tc-6B11. (**B**) Cellular binding of ^99m^Tc-6B11quantified in terms of counts per minute (CPM) in A549-CEA5-OV and A549-CEA5-KO (*n* = 3). Bars represent the mean ± SD. *** *p* < 0.001 (**C**) Flow cytometry indicating the binding of ^99m^Tc-6B11 and 6B11 to wild type A549 (A549-WT), A549 CEACAM5 knockout (A549-CEA5-KO), A549 CEACAM5 overexpression (A549-CEA5-OV) and H292 (CEACAM5 negative) cells at different VHH concentrations. Of note: data for 6B11 as control are also used in Fig. 1B

The labeling process was successful with an overall labeling yield of 73.3% (Fig. S9). The retention time of the purified product ^99m^Tc-6B11 (Fig. 2A) was consistent with that one of unlabeled 6B11 (Fig. S10). ^99m^Tc-6B11 reached a purity of 96.1% (Fig. 2A), confirmed by TLC, which indicated no activity at the furthest position of the TLC paper (data not shown) and a specific activity typically around 13 GBq/µmol. ^99m^Tc-6B11 binding to A549-CEA5-OV cells was observed and the amount of binding was positively correlated (*r* = 0.998, *p* < 0.01) with the amount of incubated ^99m^Tc-6B11, which suggested specific binding of ^99m^Tc-6B11 to A549-CEA5-OV cells. The binding of ^99m^Tc-6B11 in A549-CEA5-OV cells was on average 6.4 ± 1.7-fold higher than in A549-CEA5-KO cells, being significant (*p* = 0.0008) for 30 kBq. In addition, binding of ^99m^Tc-6B11 was attenuated (1.5 ± 0.7-fold) by using unlabeled 6B11 compared to non-blocked samples (Fig. 2B). Flow cytometry (Fig. 2C) and ELISA results (Fig. S7) indicated that specificity and affinity, respectively, remained for ^99m^Tc-6B11. The dot blotting assay confirmed specificity of ^99m^Tc-6B11 binds towards CEACAM5 (Fig. S11).

### MicroSPECT imaging of ^99m^Tc-VHH in vivo

**Fig. 3:**
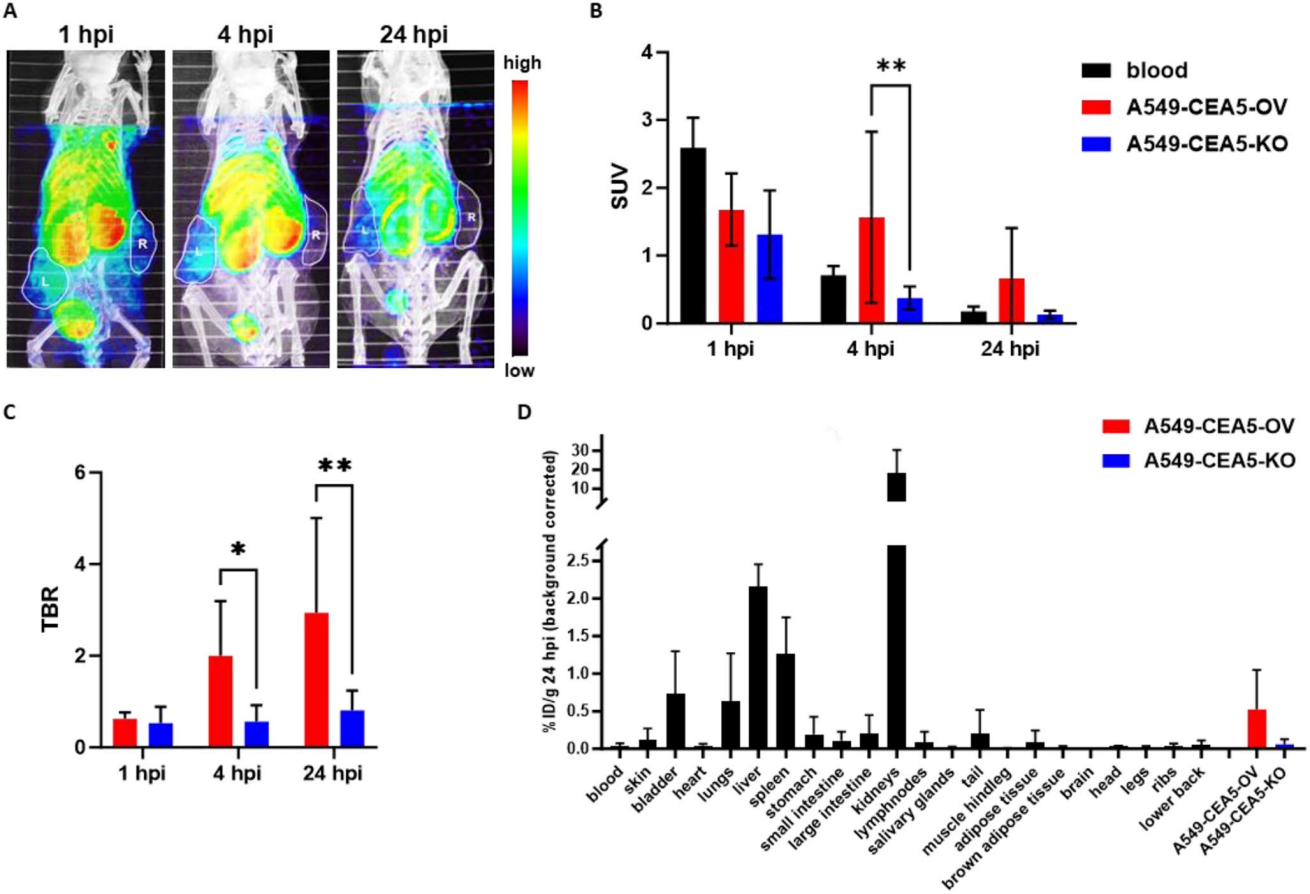
^99m^Tc-6B11 microSPECT and biodistribution. (**A**) Representative SPECT-CT images of a tumor-bearing mouse at indicated time points after ^99m^Tc-6B11 injection (left tumor: A549-CEA5-OV; right tumor: A549-CEA5-KO). ^99m^Tc-6B11 SUV (**B**) and TBR (**C**) in A549-CEA5-OV (*n* = 4) and A549-CEA5-KO (*n* = 5) at indicated time points after injection. (**D**) γ-counting results of different organs (*n* = 5) and tumors. Bars represent the mean ± SD. * *p* < 0.05, ** *p* < 0.01

The experimental setup to study the in vivo uptake of ^99m^Tc-6B11 in A549-CEA5-KO and A549-CEA5-OV tumor-bearing mice is shown in Fig. S12. Upon injection of ^99m^Tc-6B11, uptake was seen in A549-CEA5-OV tumors and in liver, kidney and bladder (Fig. 3A). From 4 hpi onwards, ^99m^Tc-6B11 SUV in A549-CEA5-OV tumors (4 hpi 1.57 ± 1.26, *p* = 0.008; 24 hpi 0.67 ± 0.75, *p* = 0.3) was higher than in A549-CEA5-KO tumors (4 hpi 0.38 ± 0.17; 24 hpi 0.13 ± 0.06) (Fig. 3B). Tumor-to-blood ratio (TBR) increased over time for A549-CEA-OV tumors, while remained constant and below unity for A549-CEA-KO tumors (Fig. 3C). At 4 hpi (*p* = 0.034) and 24 hpi (*p* = 0.003), TBR for A549-CEA-OV tumors was significantly higher than that for A549-CEA5-KO tumors. The γ-counting results confirmed these findings and demonstrated that ^99m^Tc-6B11 was predominantly renally cleared and part of ^99m^Tc-6B11 retained in the liver and spleen (Fig. 3D). ^99m^Tc-6B11 uptake was approximately 9-fold higher in A549-CEA5-OV compared to A549-CEA5-KO tumors (*p* = 0.0635, Mann-Whitney test). These results suggest that ^99m^Tc-6B11 had good ability to specifically bind with CEACAM5 in vivo.

**Fig. 4:**
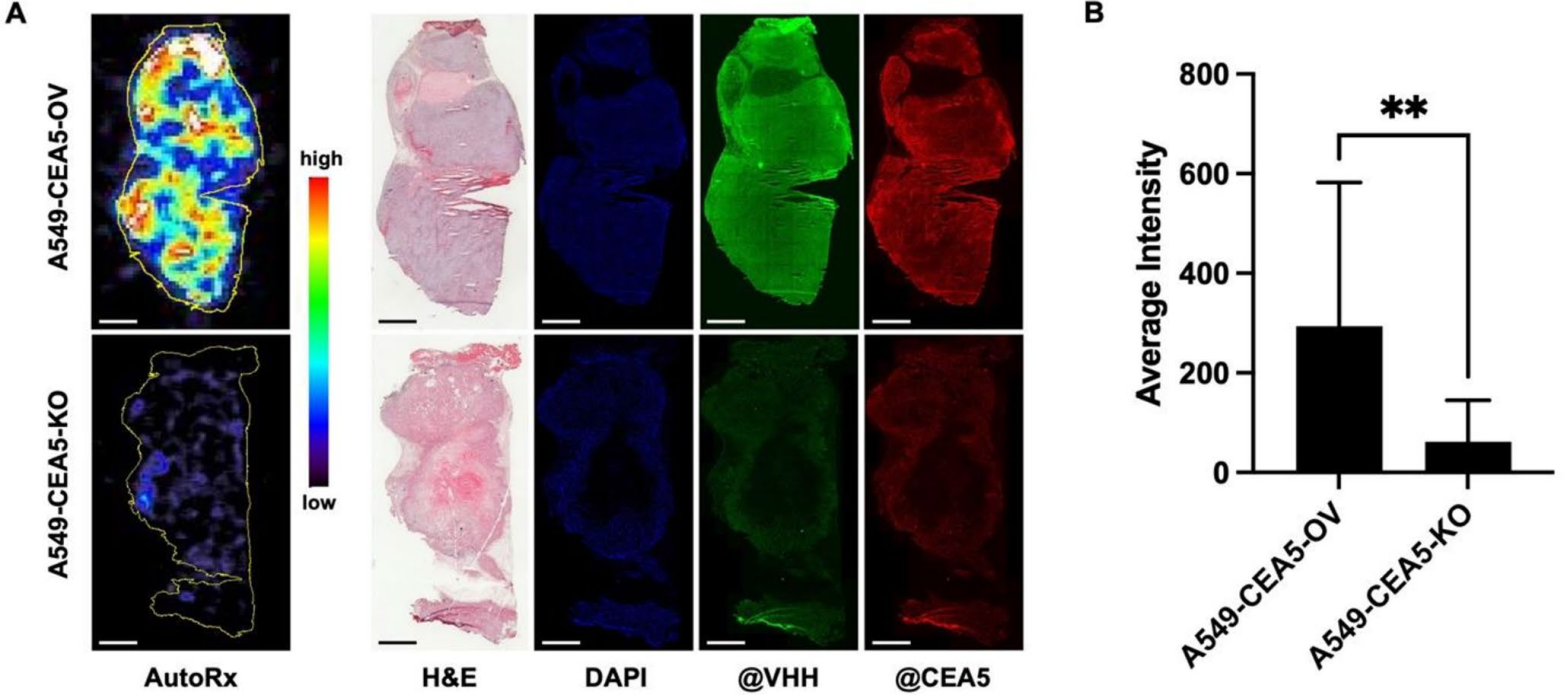
Autoradiography of ^99m^Tc-6B11 accumulation in A549-CEA5-OV tumors. (**A**) Representative autoradiography, H&E and fluorescence (DAPI: blue, alexa488-anti-VHH: green, alexa594-anti-CEACAM5: red) images. Contour (yellow) in the autoradiography images is based on the H&E staining. Scale bar = 2 mm. (**B**) Average intensity of tumor sections with SD (A549-CEA5-OV: *n* = 3, A549-CEA5-KO: *n* = 4; 6 sections per tumor). ** *p* < 0.01

In addition, autoradiography demonstrated a 5-fold higher ^99m^Tc-6B11 accumulation in A549-CEA5-OV compared to A549-CEA5-KO tumors (*p* = 0.004) (Fig. 4A and B). Immunofluorescence staining with anti-CEACAM5 commercial antibody and anti-^99m^Tc-6B11 antibody confirmed the different CEACAM5 expression levels in tumors and therefore supports the retaining of ^99m^Tc-6B11 in A549-CEA5-OV tumors (Fig. 4A).

### Anti-CEACAM5 VHH as a promising targeting moiety for VHH-based drug conjugates

**Fig. 5:**
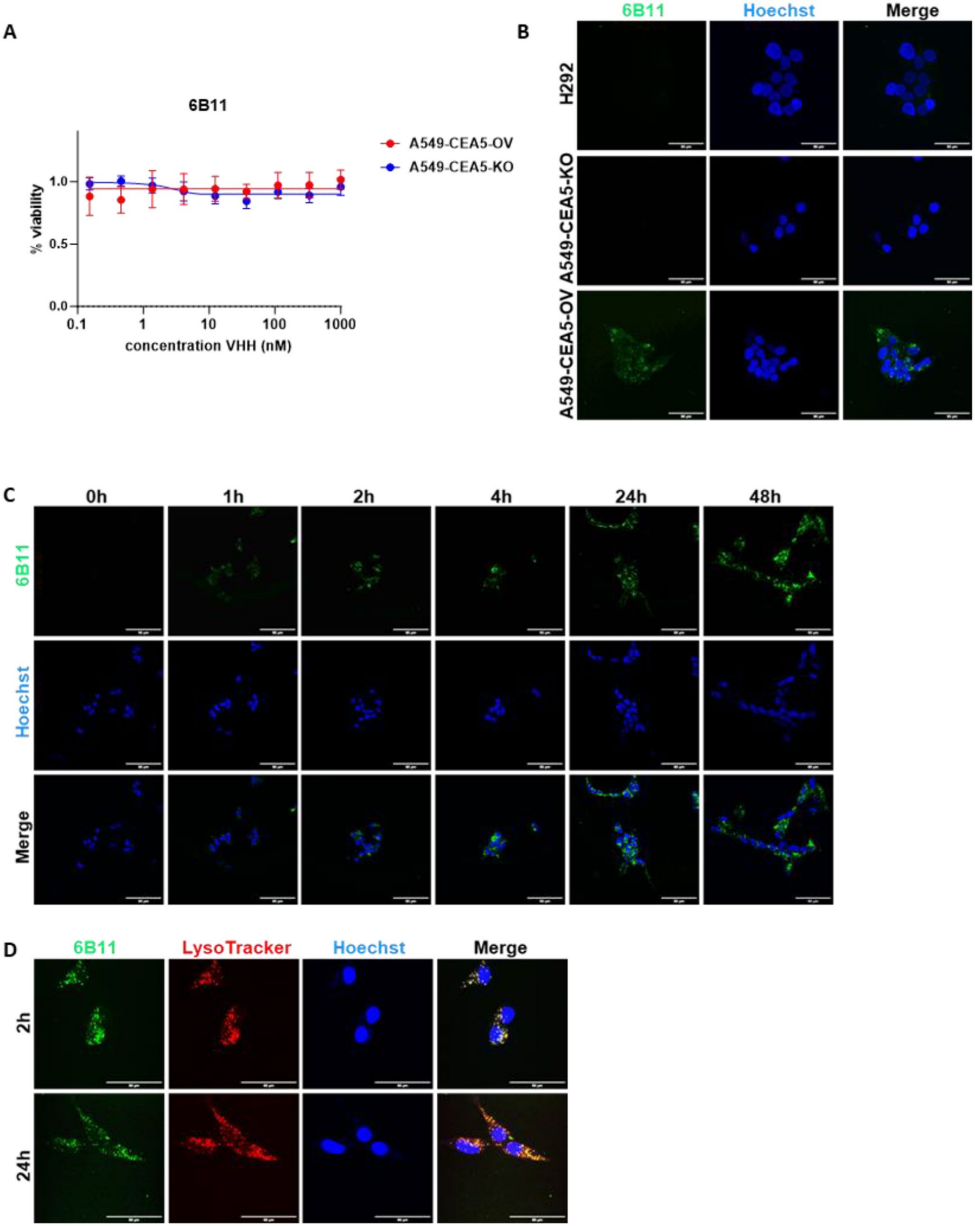
In vitro viability assay of 6B11 and cellular uptake of OG488-6B11. (**A**) A549-CEA5-OV and A549-CEA5-KO were treated with various concentrations of or 6B11 for 72 h and the viability of the cells was assessed by an Alamar Blue assay. Data represents the mean ± SD of *n* = 6 repeats. OG488-6B11internalization (green), Hoechst nuclei stain (blue) and merged image in A549-CEA5-OV, A549-CEA5-KO and H292 after 24 h accumulation (**B**) or at different time points in A549-CEA5-OV cells (**C**). (**D**) OG488-611B (green), Lysotracker (red) and merged image in A549-CEA5-OV cells after 2 h and 24 h accumulation. Scale bar = 50 μm

First, we investigated whether the developed naked anti-CEACAM5 VHHs 6B11 and 6F8 have anti-cancer activity. Overall, the results indicated that none of the VHHs could significantly inhibit cancer cell viability, migration and adhesion (Fig. 5A, S13-S15). Therefore, we investigated if 6B11 could be used as a targeting moiety for drug delivery in VHH-based drug conjugates. For this, we explored the potential of 6B11 to internalize tumor cells. Internalized OG488-6B11 was observed only in A549-CEA5-OV cells (Fig. 5B), which increased with prolonged incubation time (Fig. 5C). OG488-6B11 colocalized with lysosomes (Fig. 5D), rendering it suitable for VHH-based drug conjugates development.

## Discussion

CEACAM5 has been used as a marker for cancer diagnosis due to its overexpression in various cancers. In this study, a novel VHH against CEACAM5 was developed which was labeled with ^99m^Tc or OG488 to investigate its potential as probes for diagnostic SPECT imaging or as targeting-moiety for the development of VHH-based anti-CEACAM5 drug conjugates. We have shown that OG488-6B11 penetrates deeper and faster into spheroids compared to a conventional antibody. Our findings demonstrate the utility of ^99m^Tc-6B11 to non-invasively visualize CEACAM5-positive cancers using SPECT imaging and specific uptake has been confirmed ex vivo by γ-counting and autoradiography. Our study expands on Wang et al., who also has generated another ^99m^Tc-labeled anti-CEACAM5 VHH with good stability, however, without presenting affinity and in vivo imaging results [28]. Although therapeutic efficacy of 6B11 could not be demonstrated in the present study, we proved that 6B11 was effectively internalized in CEACAM5 positive cancer cells and accumulated in lysosomes, making 6B11 also a potential targeting moiety for VHH-based drug conjugates, which warrants further investigation.

The small molecular size enables VHHs to aggregate quickly with their target and their blood clearance is fast, which makes VHHs good candidates being developed for cancer management. ^99m^Tc-6B11 exhibited significantly higher uptake in A549 CEACAM5 overexpressing compared to knockout tumors already a few hours after tracer administration. This is in contrast with radiolabeled full-length antibodies, for which specific uptake typically takes 4–6 days, with subsequent increase in radiation exposure [30]. In the present study, high uptake of ^99m^Tc-6B11 was observed in kidney and bladder confirming renal clearance, a typical feature of VHHs [31–33] as their molecular weight is lower than the threshold that can be filtered by the glomerular membrane (< 60 kD) [34]. However, this feature of VHHs may lead to increased kidney radiation exposure, which may lead to nephrotoxicity. To reduce the risk of radiation-induced nephrotoxicity, cationic or polycationic amino acids can be applied before the administration of the radiopharmaceutical [34–36]. Furthermore, modification of the amino acid sequence in 6B11 might also reduce renal uptake [37]. ^99m^Tc-6B11 demonstrated also high liver uptake, possibly explained by its lipophilicity and the radionuclide used [38]. Altogether, to our knowledge, this is the first anti-CEACAM5 VHH that demonstrated specific tumor uptake in vivo.

The development of ADCs is based on the concept of using antibody (fragments) as a carrier to specifically bring drugs to its target cells without causing toxicity to cells which do not express the target. However, conventional antibody-based ADCs harbor many unsolved obstacles, such as low vascular leakage and solid tumor penetration rate [39]. In this study, we provided evidence that 6B11 can enter the central part of tumor spheroids, far beyond the regions reached by conventional antibodies, confirming the improved tissue penetration ability of VHHs. In addition, OG488-6B11 could be efficiently internalized and accumulated in lysosomes. Wu et al. developed a VHH-based drug conjugate based on a VHH targeting the carcinoembryonic antigen conjugated with a topoisomerase 1 inhibitor, which accumulated more effectively in tumors and inhibited tumor progression more efficiently compared with its conventional ADC [16]. Zhu et al. generated an anti-CEACAM5 VHH conjugated with monomethyl auristatin E and demonstrated that this VHH-drug conjugate could markedly inhibit tumor growth without significant toxicity [40]. Furthermore, VHHs can be combined with other antibodies that target e.g. immune cells to form cell engagers. This type of treatment can recruit immune effector cells to directly attack cancer cells, which harnesses the immune system to enhance the anti-cancer effects even if the original naked antibody is not effective [41]. Moreover, VHH can be fused with cytokines to construct immunocytokines, such as IL2 and TNF-α, for anti-cancer treatments, which can overcome the adverse effects caused by using cytokines as monotherapy [42, 43].

The VHH developed in the present study still can be improved in several aspects. Firstly, 6B11 pharmacokinetics can be improved since its fast blood clearance can result in low tumor accumulation. Several strategies have been explored to improve VHH pharmacokinetics, such as PEGylation, fusion with other functional domains, and modifying the tumor microenvironment to enhance the penetration and retention of nanobodies [44–46]. Fusing VHH with an albumin-binding domain (ABD) is a promising strategy that can extend VHH’s blood circulation time and increase tumor uptake, leading to improved imaging contrast while tissue penetration ability is little attenuated [47, 48]. Xenaki et al. evaluated an anti-HER2 VHH fused with ABD, demonstrating a 14.8-fold increased serum half-life time and a prolonged accumulation in HER2-positive xenografts, while tissue penetration remained unaffected [7]. Secondly, the potential immunogenicity of the current VHH also needs to be considered as the 6×His tag introduced during the VHH production to facilitate VHH purification and the ^99m^Tc-tricarbonyl conjugation procedure is not recommended for human applications [49, 50]. Therefore, different strategies for VHH purification and radiolabeling could be investigated, such as removing the 6×His tag after purification and applying a different chelator conjugation and radionuclide complexation [51]. Besides, humanization of VHH might also reduce the risk of VHH immunogenicity [52]. Lastly, in the present study, neither 6B11 nor 6F8 exhibited in vitro anti-cancer efficacy as monotherapy, which might be further investigated in models mimicking the tumor microenvironment (TME), as this significantly influences the efficacy of anti-cancer therapies [53]. Although no significant anti-cancer efficacy of VHHs was observed in this study, it does not rule out that the selected VHHs could exhibit different anti-tumor effects in a more complex in vivo TME given that CEACAM5 facilitates cell-cell adhesion, cell-matrix interactions including interactions with immune cells [20, 54]. Further studies should therefore explore VHHs’ efficacy in spheroids, organoids or co-cultures, or in in vivo models to better understand the potential anti-cancer effects.

## Conclusion

This study proved that the anti-CEACAM5 VHH 6B11 maintained good binding affinity and specificity after being labeled with ^99m^Tc or Oregon Green 488, which has potential of being used as a diagnostic tool for CEACAM5 positive cancers. Other applications of this VHH in molecular imaging such as labeling with PET radionuclides or other fluorescent moieties for tumor visualization can be further explored. This study also suggests that 6B11 could be used as a targeting moiety for the construction of VHH-based drug conjugates as it could be effectively internalized in CEACAM5-positive cells after binding to its target. However, due to the limited tumor uptake pattern and undesired high accumulation in kidneys and livers, modifications of the 6B11 is necessary to further improve its pharmacokinetics for developing theragnostic variants.

## Electronic supplementary material

Below is the link to the electronic supplementary material.

Supplementary Material 1

## Data Availability

The datasets generated during and/or analyzed during the current study are available from the corresponding author upon reasonable request.

## Abbreviations

VHH: Variable domain of Heavy chain of Heavy-chain-only antibody
CEACAM5: Carcinoembryonic Antigen-Related Cell Adhesion Molecule 5
SPECT: Single Photon Emission Computed Tomography
MAP-ERK: Mitogen Activated Protein - Extracellular Regulated Protein
PI3K: Phosphoinositide 3-Kinase
Akt: Protein Kinase B
MALDI-TOF-MS: Matrix-Assisted Laser Desorption Ionization Time-of-Flight Mass Spectrometry
OV: Overexpressing
KO: Knockout
OG488: Oregon Green 488
^99m^Tc: Technetium-99m
TLC: Thin Layer Chromatography
HPLC: High Performance Liquid Chromatography
ELISA: Enzyme-Linked Immunosorbent Assay
FACS: Fluorescence Activated Cell Sorter
TBR: Tumor to Blood Ratio
ADC: Antibody-drug conjugate
ABD: Albumin Binding Domain
TME: Tumor Microenvironment
SUV: Standard Uptake Value
hpi: Hour post injection
