# Supplementary material for: Development of a novel anti-CEACAM5 VHH for SPECT imaging and potential cancer therapy applications
Source: Eur J Nucl Med Mol Imaging. 2025 May 13;52(12):4569–81. doi: 10.1007/s00259-025-07321-z (PMC12491105; doi:10.1007/s00259-025-07321-z)
Supplement: Supplementary file 1 — Supplementary Material 1 [file 259_2025_7321_MOESM1_ESM.docx]

**Supplementary Materials**

**Methods**

Anti-CEACAM5 VHH identification, expression and purification

Immunization against CEACAM5 was carried out at Eurogentec S.A. in llamas SNL208, SNL209, SNL210 and SNL211. All llamas were immunized via 4 s.c injections at day 0, 14, 28 and 35 with 100 µg recombinant protein in injection 1 and 2 and 50 µg recombinant protein in injection 3 and 4. At days 0, 28 and 43, 5 mL of blood was drawn from wich the serum was used for serum titration to determine a proper immune response. To assess the presence of CEACAM5-specific antibodies, a serial dilution of the sera was tested by ELISA.

Peripheral blood lymphocytes were isolated from a large bleed at day 43 from which RNA was isolated at Eurogentec S.A., precipitated in EtOH and NaAc and dissolved in RNase-free milli-Q. The integrity of the RNA was confirmed by the clear visibility of intact 28S and 18S rRNA on a 1% agarose gel. RNA was transcribed into cDNA using reverse transcriptase and random hexamers. IG-H cDNA fragments (both conventional and heavy chain) were amplified using primers annealing at the IG-H leader sequence and the CH2 region. This PCR resulted in the amplification of two DNA fragments (~700bp and ~900bp), which represents the VHH and VH cDNA respectively. The 700bp fragment was excised, gel extracted, purified and subsequently used as a template for a nested PCR to introduce a 5` flanking SfiI restriction site. The PCR product was digested with SfiI and Eco91I, after which the digested products were ligated in frame with geneIII into the pQ81 phagemid vector. The ligated library was transformed into TG1 E.coli by electroporation. This transformation was serially diluted to determine the number of transformants, which is indicative for the library size. The total number of transformants was estimated by counting colonies in the highest dilution and using the formula: Library size = (amount of colonies) * (dilution) * 3 (mL) / 0.005 (mL; spotted volume).

For 1^st^ round selections, fresh phages were produced and subsequently precipitated. A MaxiSorp plate was coated with 5, 0.5 and 0 µg/mL recombinant CEACAM5 overnight at 4°C. For selection, 20 µL of phages was used per well. For 2^nd^ round selections, 1 µL/well of phages produced from the outputs from the 1st round are used. For both selection rounds, precipitated phages were resuspended in PBS and pre-blocked with 2% Marvel/PBS for 30 min. Pre-blocked phages were incubated with directly coated antigen for 2 h. After extensive washing with PBS-Tween, PBS-bound phages were eluted with 0.1 M TEA-solution. The phages were subsequently neutralized with 1 M Tris/HCl pH 7.5. Eluted phages were serially diluted and then used to infect TG1 cells. A serial dilution of the TG1 cells was spotted on LB -plates supplemented with 2% glucose and 100 µg/mL ampicillin and incubated overnight at 37°C.

To express the anti-CEACAM5 VHH, the sequences were PCR amplified with primers containing NdeI (cccatATGGAGGTGCAGCTGGTGGAGT) and XhoI sites (cgtactcgagttaTGAGGAGACGGTGACCTGGGTC) and ligated into the expression vector pET28b-pchG WT (a gift from Audrey Lamb, Addgene plasmid #87107). The constructed expression vector was sequenced by GeneScript with T7 and T7-Term commercial primers (T7-Forward primer: TAATACGACTCACTATAGGG, T7 term-Reverse primer: GCTAGTTATTGCTCAGCGG). The constructed expression vector was transformed into E. Coli BL12 (DE3) (NEB, C2527H). Clones were picked and incubated at 37°C at 200 rpm in 5 mL LB culture medium containing kanamycin (100 µg/mL, Sigma-Aldrich, K1377) overnight. The next day, 50 µL bacterial culture was transferred into 5 mL fresh medium, grown till OD_600_ reached 0.6~1.0 and stored at 4°C overnight. The day after, 2 mL bacterial culture was centrifuged (6000 rpm, 1 min, room temperature (RT)) and the bacterial pellet were resuspended in 2 mL fresh 2xYT medium (Sigma-Aldrich, Y2377), transferred into 100 mL 2xYT medium, grown till the OD_600_ reached 0.5~1.0 followed by IPTG (Sigma-Aldrich, I6758) induction culture for 5.5~6 h. Afterwards, the bacterial pellet for purification was harvested by centrifugation (15 min, 3000 rpm, 4°C).

To obtain VHH, the bacterial pellet was lysed using BugBuster® Master Mix (Merck, 71456). HisPur™ Ni-NTA Spin Purification Kit (Thermofisher, 88229) was used for VHH purification according the manufacturer’s procedure. Imidazole in VHH was removed using Zeba™ Spin Desalting Columns (Thermofisher, 89894) or Pierce™ Protein Concentrators (Thermofisher, 88526). VHH yield was determined using Pierce™ BCA Protein Assay Kits (Thermofisher, A55864).

Coomassie Staining

The products from each purification step were separated using SDS-PAGE electrophoresis and stained with Coomassie staining buffer (0.01% (w/v) Brilliant Blue, 25% (v/v) Isopropylalcohol, 10% (v/v) glacial acetic acid, 65% (v/v) MilliQ water) at RT for 1 h. Gels were destained with destaining buffer (40% (v/v) ethanol, 10% (v/v) glacial acetic acid, 50% (v/v) MilliQ water) by washing on a horizontal shaker (50~60 rpm, 3 times, 15 min, RT) and washed with water on a horizontal shaker (50~60 rpm, RT) overnight. Gels were imaged by Azure imaging system (bright field mode).

Western Blot

Cells in exponential growth phase were placed on ice and rinsed twice with pre-cooled PBS. Upon addition of an appropriate volume of RIPA lysis buffer containing protease inhibitors, cells were detached using a cell scraper, collected into an Eppendorf tube and incubated on ice for 30 min. After three freeze (liquid nitrogen) – thaw (37°C water bath) cycles, samples were centrifuged (> 10000 rpm, 15 min, 4°C) and supernatant was collected into a new Eppendorf tube. Sample protein concentration was determined using the Bradford assay (Bio-Rad). The prepared samples were stored at -80°C. Proteins, separated using SDS-PAGE gel electrophoresis (8% running gel - 15% stacking gel), were transferred to a nitrocellulose membrane (NC membrane). Afterwards, the membrane was blocked using 5% fat-free milk (w/v in PBST) at RT for 1.5 h and probed overnight using a rabbit anti-CEACAM5 monoclonal antibody (1:1000, ab133633, Abcam) and mouse anti-β-actin antibody (1:1000, 08691001, MP Biomedical) at 4°C. Subsequently, the membrane was washed and probed using a goat-anti-rabbit-HRP antibody (1:10000, #7074) and goat-anti-mouse-HRP antibody (1:10000, #7076), both from Cell Signaling Technology. Signal was detected using ECL Prime Western Blotting reagents (Cytiva, GERPN2232) and imaged with an Azure imaging system.

ELISA for testing VHH binding affinity

A 96 well plate (M9410, NUNC) was coated with 1 µg/mL human recombinant CEACAM5 protein (11077-H08H, Sino Biological) at 4°C overnight. The next day, the 96 well plate was washed 3 times with PBST (0.1% Tween20 in PBS) to remove unbound antigen. After blocking with 2% BSA (in PBS; 100 µL/well; 2 h at RT), the plate was washed 3 times with PBST and incubated with serial diluted VHH (100 µL/well; 2 h at RT). Upon primary antibody incubation, the plate was washed 5 times with PBST to remove unbound antibody and incubated with an HRP labelled anti-VHH antibody (1:1000; 128-035-003, Jackson 1 h at RT). After incubation with antibody, the plate was washed 5 times with PBST and incubated with 100 µL/well TMB substrate (421101, Biolegend) and subsequently 100 µL/well stop solution (Biolegend, 423001) to stop the reaction. OD_450_ was measured using a SpectraMax ID3 Multi-Mode Microplate Reader.

Flow cytometry for testing VHH cellular binding

Target cell suspension (10^5^ cells in 100 µL serum free RPMI1640 per well) was added to a U-bottomed 96 well plate (Corning, 7007). 100 µL/well pre-diluted primary antibody (final concentration 1 µg/mL or 0.33 µg/mL), i.e. 6B11, ^99m^Tc-6B11 (after complete decay) and OG488-6B11 in 1% BSA- RPMI1640 medium, was mixed with the cell suspension and incubated at 4°C for 1 h. After incubation, cells were washed 3 times with pre-cold PBS to remove unbound antibody, resuspended and incubated with 200 µL/well alexa488 labeled secondary antibody (1:1000; 128-545-160, Jackson) at 4°C for 30 min. The cells were washed with pre-cooled PBS and resuspended in 200 µL 1% PFA. VHH binding was measured with Flow Cytometer using FACS Diva 6.1.1 software (BD, FACS Canto II). The results were analyzed by using FlowJo V10. FSC-A vs. SSC-A was used for gating the targeting cells and FSC-H vs FSC-W was used for gating single cells. The cells in the single cell gate were used for alexa488-A analysis.

ICC for testing VHH binding specificity on cells

Cells were seeded in a chamber slide (80841, Ibidi GnbH) so 60%~80% confluency was reached on the next day and cultured overnight to let the cells attach to the bottom properly. The next day, culture medium was discarded and cells were washed 3 times with PBS, fixed with 4% PFA (v/v in PBS) for 15 min and blocked using 5% goat serum for 20 min at RT. VHH (1 µg/mL) was added to each well and cells were incubated at 4°C overnight. Cells were washed 3 times with PBST (PBS with 0.05% Tween-20) and incubated with rabbit-anti-VHH antibody (1:500, QE19, QVQ) at 37°C for 1 h. After 3 times washing with PBST, cells were incubated with alexa488 goat-anti-rabbit IgG (H+L) (1:500, A-11008, Thermofisher) 1 h at RT. After PBST wash and blocking with 5% rabbit serum for 20 min at RT, cells were incubated with AffiniPure Fab Fragment goat-anti-rabbit IgG (H+L) (111-007-003, Jackson) at 1:50 dilution for 1 h at RT. Then cells were washed with PBST 3 times, incubated with rabbit-anti-CEACAM5 antibody (1:500, ab133633, Abcam) for 1 h at 37°C, washed 3 times with PBST and incubated with alexa594 goat-anti-rabbit (1:500, A11012, Thermofisher) for 1 h at RT. Cell nuclei were stained with Hoechst 33342 (1 µg/mL) after 2 times PBST and one PBS washing step. The slide was sealed after mounting cells with fluorescence mounting medium (S3023, Dako). Images were taken with a Leica SPE confocal microscope at 400× magnification. The images were thresholded just above the background to determine the positive staining area and the intensity of the fluorescent signal was determined. The coefficient of similarity (DICE) was calculated according to $\frac{2\times overlaping area of target and VHH}{target express area+VHH binding area}$. The target express area was the area stained positive by the commercial anti-CEACAM5 antibody, the VHH binding area was the areas stained positive by the VHH. The closer coefficient to 1, the better specificity the candidate VHH had.

Synthesis of OG488-6B11

A lyophilized 6B11 stock (1.31 mg) was dissolved in 170 µL buffer containing 150 µL HP-buffer (HEPES+NaCl, pH 7.4) and 20 µL MeCN. An Oregon-green-488 succinimide (O6149, Invitrogen) stock (3 mg/mL) solution was prepared by adding dimethylformamide (DMF) to the reagent bottle. 1 µL Dipea and 3 eq OG488 stock solution were added to 6B11 and the reaction was monitored in MALDI-TOF-TOF-MS in mid mass mode after diluting the sample 50 times with 50% H_2_O / 50% MeCN (v/v) containing 0.1% TFA and 1:1 (v:v) mixed with α-Cyano-4-hydroxycinnamic acid (HCCA) matrix. The reaction was monitored over 3 h, 1 eq OG488 was added each hour until all the VHH was labeled with at least 1 OG488 moiety. Afterwards, the OG488-6B11 was purified on a reverse-phase analytical HPLC with a C4-column for protein separation. Buffer A (0.1% TFA in H_2_O) and buffer B (0.1% TFA in MeCN/H_2_O (9/1, v/v)) were used as mobile phase. A UV-detector at 220 nm was used to measure the absorbance of the peptide bounds.

Spheroid Formation and VHH diffusion assay

Single cells (A549-CEA5-OV 500 cells/well, A549-CEA5-KO 3000 cells/well, H292 2000 cells/well) were seeded in an ultra-low attachment plate (7007, Corning) and plates were incubated at 37°C / 5% CO_2_ for minimally 3 days to enable spheroid formation. Upon reaching a diameter of approximately 500 µm, spheroids were incubated at 37°C / 5% CO_2_ for 1, 3 and 24 h with culture medium containing 25 nM of OG488-6B11 or Alexa488-anti-CEACAM5 antibody [EPCEAR7] (ab214868, Abcam). After incubation, medium was removed, spheroids were rinsed twice with PBS and fixed by submersion in 2% (v/v) PFA for 30 min followed by 4% (v/v) PFA for 30 min and 100 mM glycine-PBS solution for 30 min at RT to remove background fluorescence. Then spheroids were washed three times with PBS. Images were taken with 40× magnification using a Leica SPE confocal microscope with z-stack mode and quantified according to the description in the study of Xenaki et al. (2021). The thresholded area that was the area covered by the diffusing fluorescently labelled VHH/full-length antibody and the total spheroid area were used to calculate the displacement of VHH/full-length antibody. The area of the whole spheroid and the unstained area was measured and the radius of these two areas (r_total_ and r_unstained_) were calculated according to $A=\pi r^{2}$. The covered radius was calculated according to $R\text{=}r_{\mathrm{total}}-r_{\mathrm{unstained}}$_._ The percentage of spheroid’s covered radius was calculated according to $\frac{R}{r_{\mathrm{total}}}\times100\%$.

Radiolabeling VHH with ^99m^Tc

^99m^Tc (±2 GBq) was prepared in the GMP lab. The IsoLink kit for tricarbonyl (Center for Radiopharmaceutical Sciences) was pre-flowed with N_2_ to remove all O_2_ from the vial. The ^99m^TcO_4_^-^ solution was added to the IsoLink (Na_2_B_4_O_7_+Na_2_BH_3_CO_2_+NaKC_4_H_4_O_6_, pH=11) and incubated in an oil-bath at 100°C for 20 min. The product was transferred to a new reaction vial and physiological pH was reached by adding 0.1 mL of HCl. 50~60 µg of the His-tagged VHH 6B11 was added to the vial containing neutralized ^99m^Tc(CO)_3_^+^. The mixture of reagents was incubated in a water bath at 37°C under a gentle flow of N_2_ gas. for about 2 h till the volume in the vial reduced to less than 500 µL. The product was purified using ultrafiltration tubes (UFC5003, Millipore).

^99m^Tc-VHH quality control assessment was performed using TLC and HPLC

10 cm long strip of ITLC-SG paper (Agilent) was used as carrier for TLC. A drop of 5 µL radioactive synthesis product was added at the bottom of the TLC paper and its end was placed in 0.1 M citrate buffer. The solvent was allowed to reach up to 2 cm before the end of the TLC paper, the paper was cut into 3 equal-sized pieces and radioactivity of each piece was measured using a dose calibrator (CRC-25R, CAPINTECH.Inc). For HPLC, Aeris Widepore XB-C18 column (dimensions 2540x4.6 mm, pore size 3.6 µm, Phenomenex) was used. As mobile phase, we used a gradient: 0-3 minutes: 100% 0.1% TFA in H2O, 3-23 minutes: linear gradient going from 0 to 100% 0.1% TFA in acetonitrile, 23-24 minutes: 100% 0.1% TFA in acetonitrile, 24-30 minutes: linear gradient going back to 100% 0.1% TFA in H2O. The signal was measured by γ-counting, expressed as count per second (cps).

^99m^Tc-6B11 *in vitro* binding and blocking assay

Cells were cultured in a 24 well plate (4×10^4^ cells/well) overnight and washed twice with pre-cooled PBS the day after. A serial dilution (30, 10 and 3.33 kBq representing 2300, 768 and 256 nM VHH respectively) of ^99m^Tc-6B11 was added to each well and incubated on ice for 1 h. Then the cells were washed 3 times with pre-cooled PBS. Cells were lysed with 1 mL/well 1 M NaOH (5 min, RT) and all cell lysis was collected into tubes to measure radioactivity by γ-counting (PerkinElmer). The radioactivity, expressed in counts per minute, was normalized to the number of cells determined by crystal violet staining in a parallel plate. For the blocking assay, cells were first incubated with 50 µg non-radiolabeled 6B11 (1 h on ice) before adding ^99m^Tc-6B11.

*In vivo* study to test ^99m^Tc-VHH binding specificity and biodistribution in tumor-bearing mice

All animal experiments were in accordance with local institutional guidelines for animal welfare and approved by the Animal Ethical Committee of the University of Maastricht (AVD10700202216527). Mycoplasma-free A549-CEA5-KO cells (1.5×10^6^ cells in 50 µL matrigel, Corning, 354234) were injected subcutaneously in the right flank of eight-week-old female Crl:NU-Foxn1-nu mice (Charles River). When palpable tumors were formed, A549-CEA5-OV cells (1.5×10^6^ cells in 50 µL matrigel) were injected subcutaneously in the contralateral flank. Tumor growth was monitored using a Vernier caliper by measuring tumors in three dimensions for a maximum of 5 times per week. Tumor volume was calculated using the formula: $\text{tumor volume=}\frac{\text{(a-0.5 mm)(b-0.5 mm)(c-0.5 mm)π}}{\text{6}}$ , where a, b and c are the three dimensions of the tumor and 0.5 mm being a correction for the thickness of the skin. When tumor volume reached 623 ± 339 mm^3^, mice were intravenously injected with ^99m^Tc-6B11 (64.9±21.5 MBq) under isoflurane inhalation anesthesia (4% induction, 2.5% maintenance). microSPECT (U-SPECT, MILabs B.V.) image acquisition was performed under isoflurane anesthesia at 1 h (duration of scan: 15 min), 4 h (duration of scan: 30 min) and 24 h (duration of scan 1 h) post tracer injection with 8 positions spiral scanning mode using a rat collinator. microSPECT images were reconstructed using U-SPECT (Rev 2.0) (photopeak window: 126.0 - 154.0 keV; voxel size 0.4 mm; number of subsets 16; number of iterations 6; apply post filter: yes). After each microSPECT acquisition, a high-resolution microCT image was acquired using the X-RAD 225Cx (Precision X-Ray Irradiation Inc.) with following settings: 80 kVp, 500 mAs protocol, 2 mm aluminum filter, acquisition rate 5 frames/second gantry rotation of 1 revolution/min, while the animal remained positioned in a clickable bed. Images were reconstructed using Feldkamp’s filtered back-projection (Smart+ software, v1.20.8.2; Precision X-Ray Irradiation Inc.). The CT dose to skin was 39 cGy. SPECT and CT images were co-registered using AMIDE software (version 0.9.0; Loening et al Mol Imaging 2003). For each dataset, 3-dimensional regions of interest (ROIs) were manually drawn over the tumors and the heart on the CT image. The ROIs were transferred to the respective SPECT image and mean activity data (in Bq/mL) were obtained. All data were corrected for ^99m^Tc decay toward injection and mean standardized uptake values (SUV_mean_) were calculated. Additionally, tumor-to-blood uptake ratios (TBR) were calculated as the ratio of the SUV_tumor_ and the SUV_heart_.

After the last imaging session, mice were killed by cervical dislocation. Tumors and organs were excised for γ-counting. Tissues and tumors were weighted and disintegrations for each sample were collected over 1 min using an energy window between 98 and 187 keV (^99m^Tc protocol, window 98-187, WIZARD^2^ Perkin Elmer). Radioactivity was corrected for decay, cross-calibrated to the SPECT using injection standards and uptake was calculated as the percentage injected dose per gram of tissue (%ID/g). Part of the tumor was snap-frozen and 10 μm sections were cut (CM1860, Leica) for autoradiography followed by consecutive 7 μm sections for histological validation. The autoradiography sections were exposed overnight to a FujiFilm BAS-SR2040, the film was scanned by a Typhoon FLA 7000 (GE Healthcare) in phosphorimaging mode (IP filter, 650 mm laser) and images were quantified by ImageQuantTL (8.1). Tumor areas were circled manually, and the average intensity per surface area was analyzed automatically. The intensity of signal in background was deducted from that in tumor area.

The sections for histological validation were air-dried, fixed in aceton (4°C) and washed with PBST. Next, non-specific binding was blocked using 5% (v/v) normal goat serum in PBST for 30 min at RT. Sections were incubated overnight at 4°C with alexa488 goat-anti-VHH (1:250, Jackson) and rabbit-anti-CEACAM5 antibody (1:1500, ab133633, Abcam) in antibody diluent (Agilent Technologies). The next day, sections were washed with PBST (3x5 min, RT) and incubated for 1 h at RT with alexa594 goat-anti-rabbit (1:1000, A11012, Thermofisher) in antibody diluent. Cell nuclei were stained with DAPI (1 µg/mL in PBS, 2 min at RT) after 2 times PBST and one PBS washing step (5 min, RT). The slide was sealed after mounting with fluorescence mounting medium (S3023, Dako). Images were acquired using an Olympus BX51WI fluorescence microscope equipped with a Hamamatsu EM-CCD C9100 digital camera, a motorized stage (Ludl Mac 2000) and a 10× magnification objective. Following exposure times were applied: DAPI channel - 40 ms/field, 488 channel - 800 ms/field, 594 channel - 300 ms/field. Micromanager v2.0.3 software was used for automated image acquisition and stitching of images. The images were thresholded above the background to determine the positive staining area.

Coverslips were detached in milliQ, followed by incubation with Mayer’s hematoxylin (J. T. Baker, 10 min, RT), tap water (10 min, RT), milliQ (5 min, RT) and eosin (J. T. Baker, 1 min, RT). Upon incubation with increasing concentrations ethanol, slides were air-dried and closed with a new coverslip in xylene. Next, slides were scanned (x20) using a Precipoint M8 whole slide scanner, converted into tiff-files and resized using FIJI software. Section contours were obtained by creating a mask upon thresholding above background and transferred to the autoradiography images.

Viability Assay

Optimized number of cells (A549-CEA5-KO 1500 cells/well, A549-CEA5-OV 500 cells/well) diluted in 100 µL growth medium were seeded into 96-well plate and cultured overnight. The next day, 100 µL culture medium containing 2× the final concentration of VHH was added to each well (final concentration from 1 µM to 0.05 by serial dilution). The cells were cultured continuously for 72 h. Culture medium was removed and 100 µL/well 10% AlamarBlue solution (DAL1100, Invitrogen) was added. The cells were incubated at 37°C for 2 h and the fluorescence at 570 nm was measured using a SpectraMax ID3 Multi-Mode Microplate Reader.

**Wound healing Assay**

Cells were seeded into 35 mm dishes with a culture insert (80466, Ibidi) and incubated at 37°C overnight to let cells attach properly. The next day, the culture insert was removed from the 35 mm dish with a confluent cell culture, making a wound, and 2 mL serum-free culture medium containing 1 µM VHH was added to the dish. In each dish, 4 wounds were imaged and the percent remaining of the original wound area was calculated. Images were taken using a Nikon Eclipse Ts2 microscope with 40× magnification at 0 h, 24 h, 32 h and 48 h after adding VHH. The wound area was analyzed using FIJI software. The percentage of remaining area was calculated as follows: $\text{\% remains of origin=}\frac{\text{wound area at different time point}}{\text{original wound area}}\text{×100\%}$.

Adhesion Assay

A 96-well plate was coated with human fibronectin (100 µL of 10 µg/mL stock; RP-43130, Thermofisher) at RT for 1 h. Then liquid was aspirated and the plate was washed 3 times with PBS. The plate was blocked with 1% (v/v) BSA in serum-free culture medium at 37°C for 1 h. Afterwards, the blocking buffer was discarded and 5×10^4^ cells were added into each well together with different VHH concentrations (1 µM or 2 µM). Cells were incubated at 37°C for 2 h and washed intensively 5 times with PBS. In parallel, selected wells were not washed and used as total cell input. After the washing steps, 100 µL/well 10% Alamar Blue working solution was added into each well and incubated at 37°C for 2 h. The fluorescence at 570 nm was measured with a SpectraMax ID3 Multi-Mode Microplate Reader. The adhesion ratio was calculated with the formula: $\text{adhesion ratio=}\frac{\text{absorbance}\left( \text{washed wells} \right)}{\text{absorbance}\left( \text{unwashed wells} \right)}$.

**Supplementary figures**

**
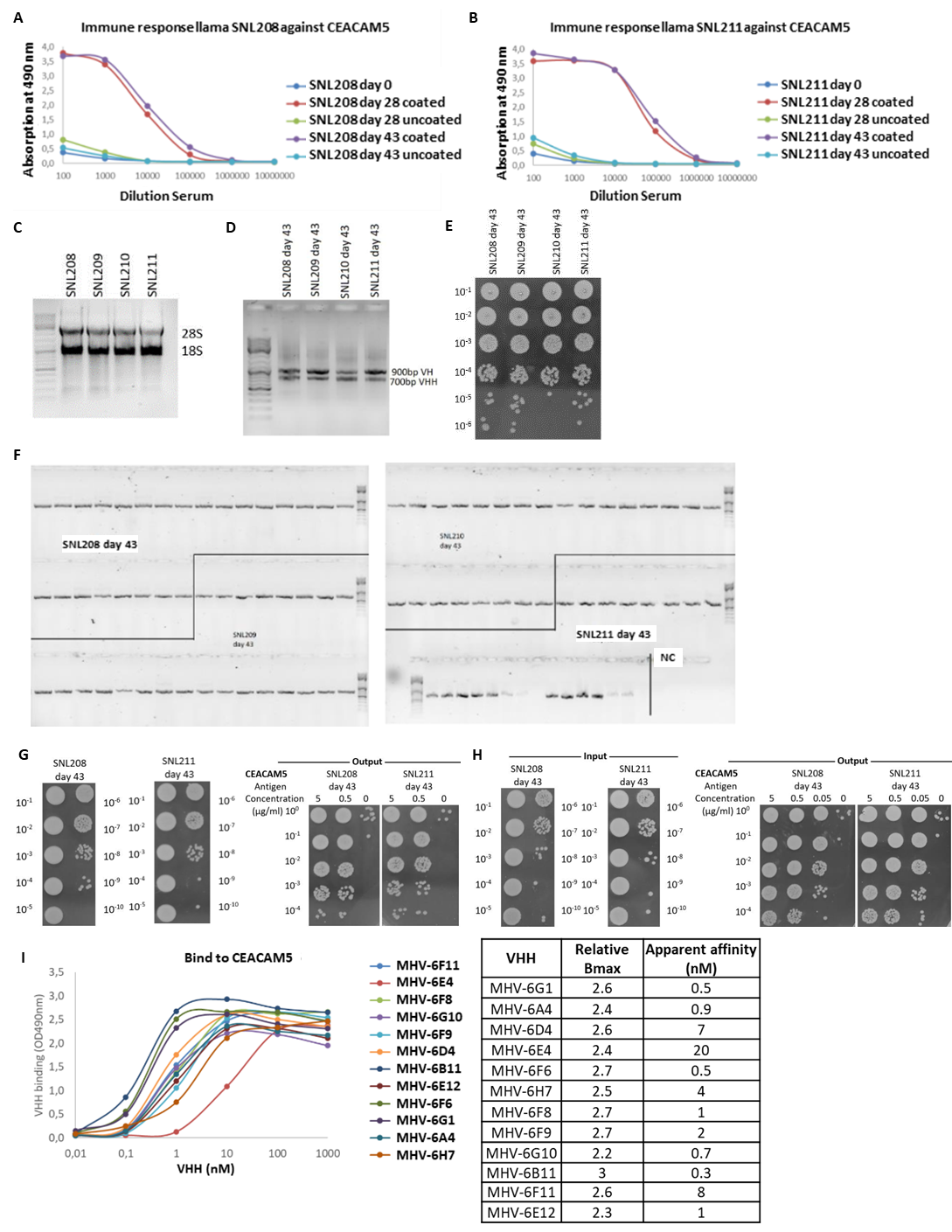
**

**Figure S1.** **VHH screening from phage display library.** Immune response of llama SNL208 (A) and SNL211 (B) to immunization with human CEACAM5 protein. The bound VHHs were detected by rabbit-anti-VHH followed by donkey-anti-rabbit-HRP. (C) The quality of whole extracted RNA from different llamas was confirmed on a 1% agarose gel. The 28S and 18S rRNA bands are indicated. (D) The heavy chain fragments of llama immunoglobulin were amplified. The cDNA of VHH was indicated as the 700 bp band. (E) VHH cDNA fragments were cloned into phagemid and transformed into E. coli TG1. (F) Confirmation of the VHH library by colony PCR. (G) and (H) Results of the first and the second round selection on coated CEACAM5 respectively. (I) Binding of purified selected VHHs to immobilized CEACAM5 by ELISA.


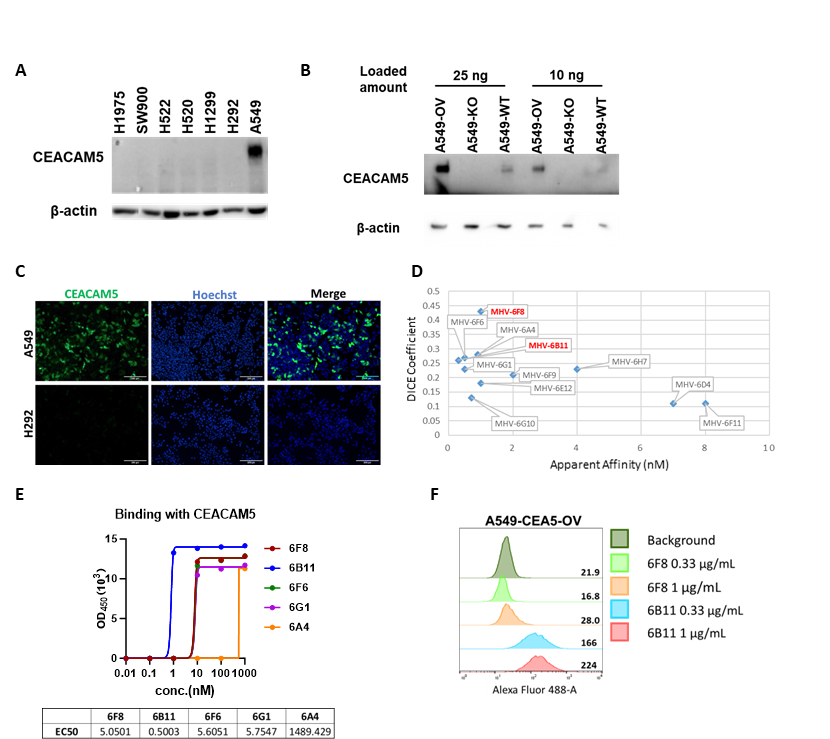


**Figure S2. Selection of the appropriate cancer models and lead VHH.** (A) CEACAM5 protein expression levels in different tumor cell lines assessed by Western blotting. Actin served as loading control. (B) CEACAM5 expression levels in A549 CEACAM5 overexpressing cells (A549-CEA5-OV), A549 wild type cells (A549-WT) and A549 CEACAM5 knockout (A549-CEA5-KO) cells assessed by Western blotting. Actin was used as loading control. (C) CEACAM5 staining (green) in A549 and H292 cells determined by ICC using a commercial CEACAM5 monoclonal antibody. Hoechst 33342 staining shows cell nuclei (blue). Scale bar=200 µm. (D) Relationship between DICE similarity coefficient and apparent affinity for 11 VHHs candidates. (E) VHH recombinant protein binding affinity evaluated by ELISA for 6 VHHs. (F) Mean fluorescence intensity of 6F8 and 6B11 bound to CEACAM5 overexpressing A549 (A549-CEA5-OV) cells depending on the concentration of the VHH in the medium.


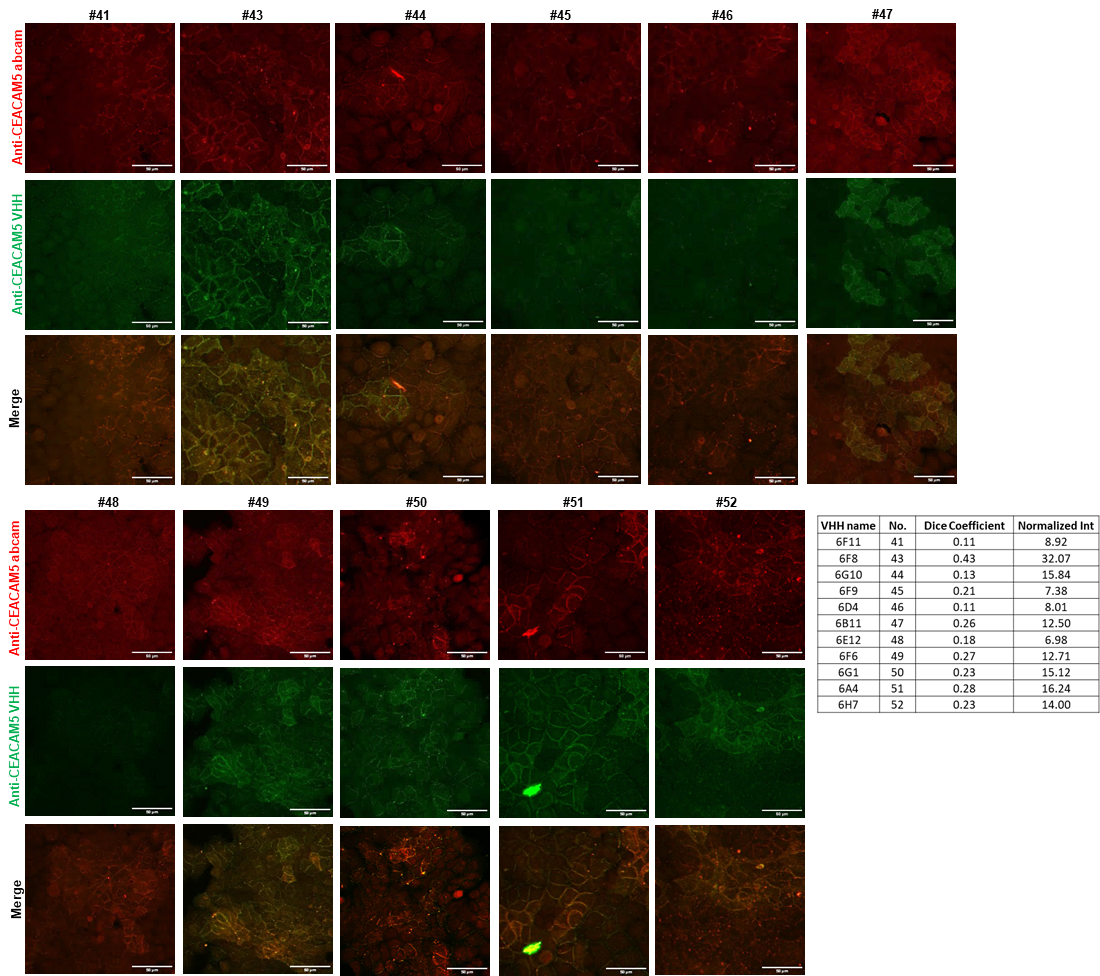


**Figure S3.** **VHH cellular binding affinity.** A549 wild type cells were co-stained with the anti-CEACAM5 VHHs (green) and the commercial conventional anti-CEACAM5 antibody. To reduce the bias during the analysis, each VHH was anonymized by given a number. The DICE similarity coefficient was calculated. The normalized fluorescence intensity (Normalized Int) was the results of normalizing the raw fluorescence intensity with the number of cells.


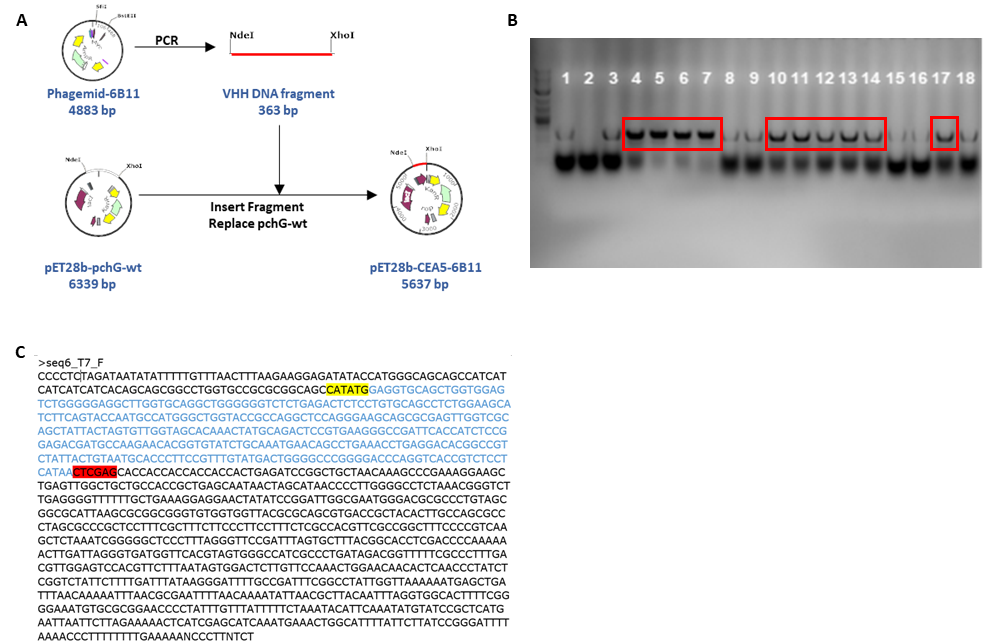


**Figure S4.** **Construction of pET28b-6B11 plasmid for VHH production.** (A) The general process of plasmid construction, exemplified by the construction of pET28B-CEA5-6B11. (B) Colony PCR of transformed E. Coli NEB Stbl to identify colonies with correct insertion size. Colonies indicated with red boxes may contain correctly inserted pET28B-CEA5-6B11.


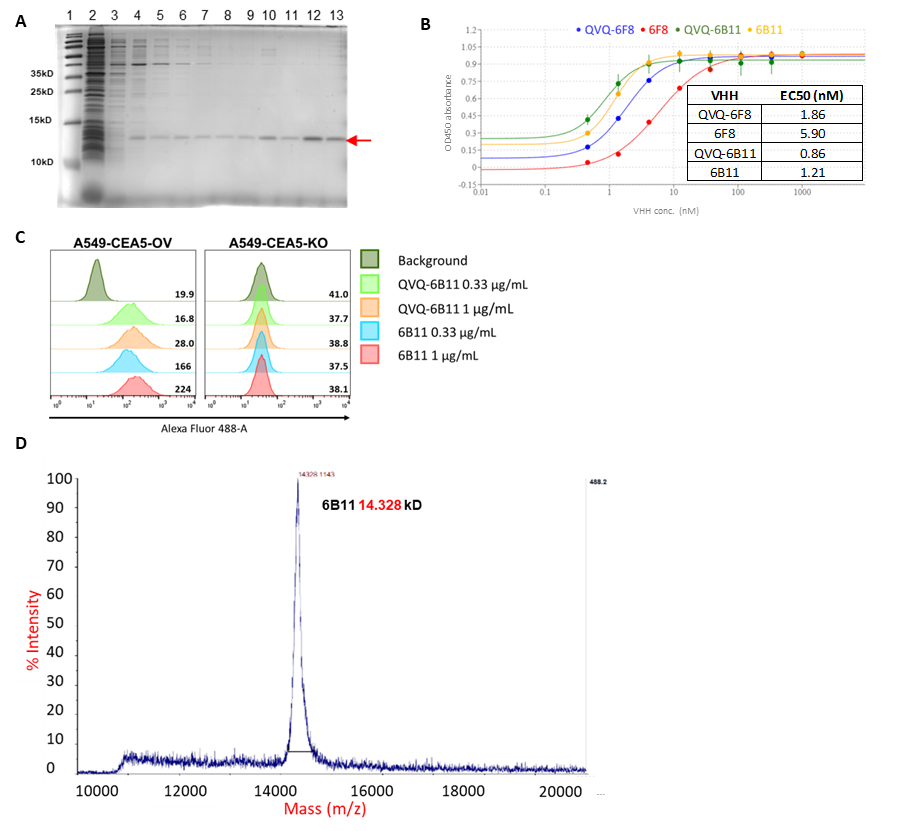


**Figure S5.** **Purification and characterization of anti-CEACAM5 VHH 6B11.** (A) Coomassie staining of the different purification fractions. Lane 1: protein marker; lane 2: induced total bacterial protein, lane 3-13: fractions collected from the Ni^+^-NTA column by washing with increasing concentrations of imidazole (lane 3: 25 nM, lane 4-5: 40 nM, lane 6-7: 70 nM, lane 8-9: 80 nM, lane 10-11: 100 nM and lane 12-13: 250 nM). VHH 6B11 is marked by the red arrow. (B) ELISA apparent affinity of in house produced VHHs compared to QVQ produced VHHs. (C) Cellular binding at different VHH concentration in CEACAM5 overexpressing A549 (A549-CEA5-OV) and CEACAM5 knockout A549 (A549-CEA5-KO) NSCLC cells tested by flow cytometry. (D) Molecular weight of 6B11 VHH, calculated based on results of MALDI-TOF-TOF-MS.


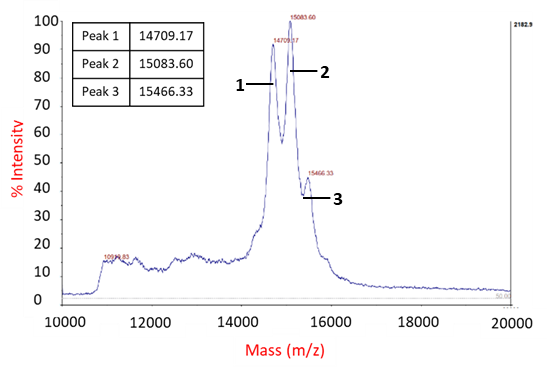


**Figure S6. Synthesis of fluorescent labeled VHH.** The molecular weight of the products formed during fluorescent labeling process of 6B11 VHH with OG488 dye was assessed by MALDI-TOF-TOF-MS.


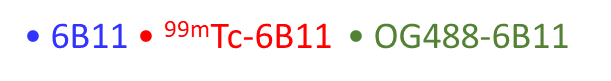

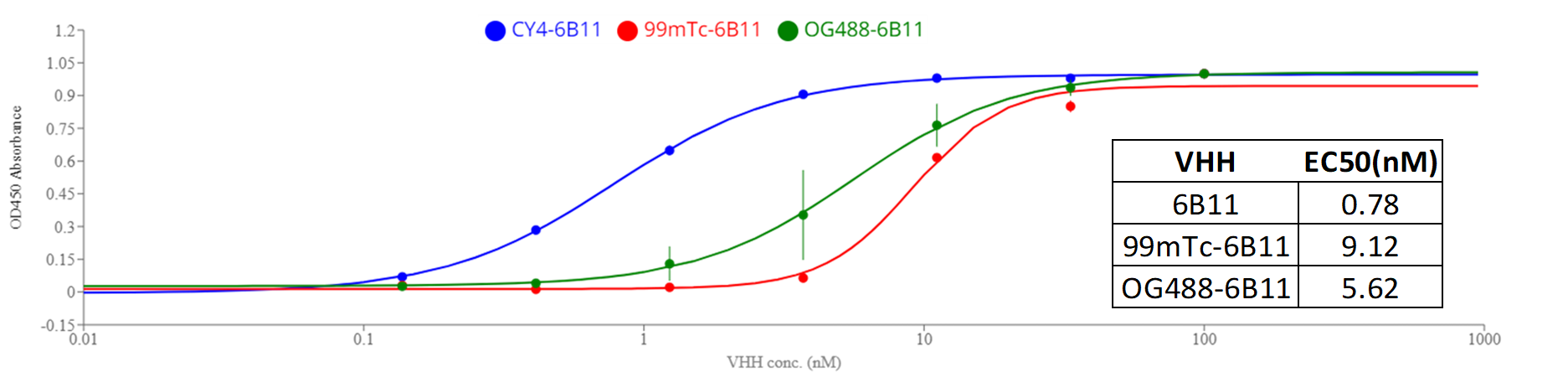


**Figure S7.** Apparent affinity of 6B11,^99m^TC-6B11 and OG488-6B11 by ELISA assay.


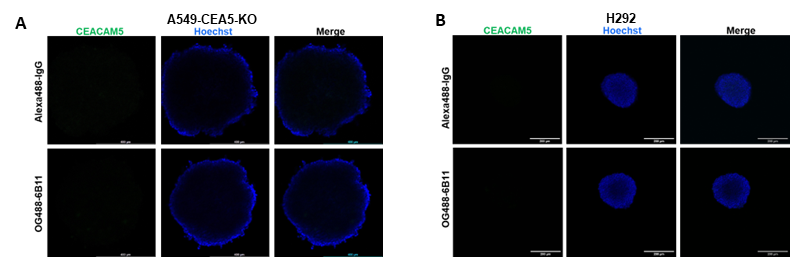


**Figure S8.** Distribution of fluorescent anti-CEACAM5 Alexa488-IgG and OG488-6B11 (green) in A549-CEA5-KO and H292 spheroids. Cell nuclei (Hoechst 33342) is shown in blue.


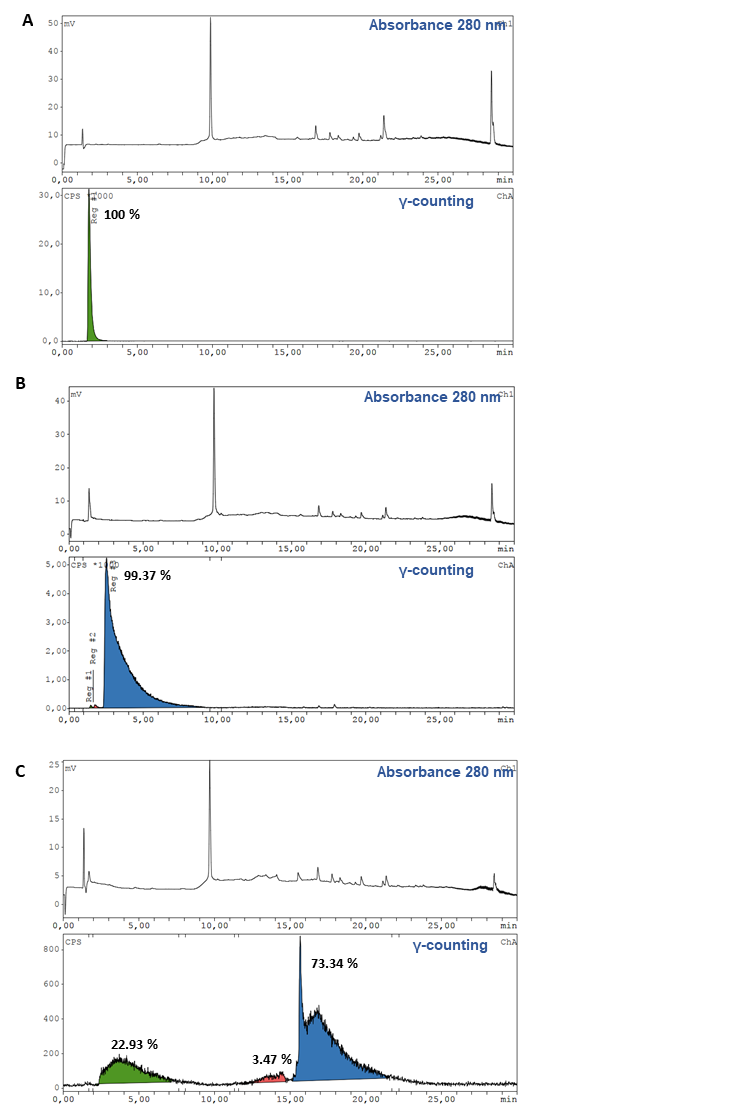


**Figure S9. Monitoring the radiolabeling process of ^99m^Tc-6B11.** HPLC result of testing ^99m^TcO_4_^-^ (A), ^99m^Tc(CO)_3_^+^ (B) and unpurified ^99m^Tc-6B11 (C) at 280 nm absorbance and gamma counting.


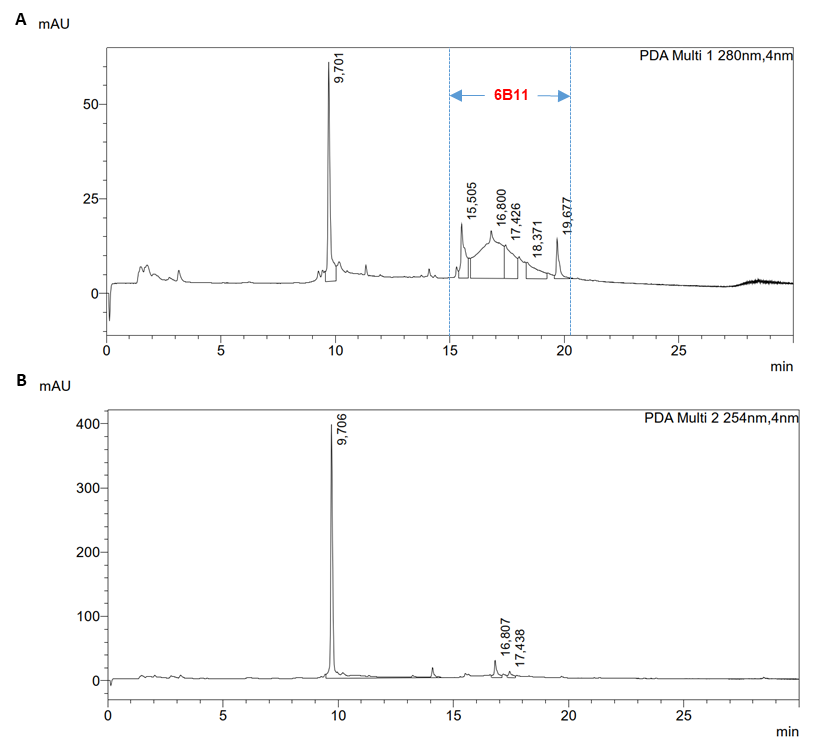


**Figure S10. Verification of anti-CEACAM5 VHH used for radiolabeling.** (A) HPLC result for testing 6B11 at (A) 280 nm and (B) 254 nm.


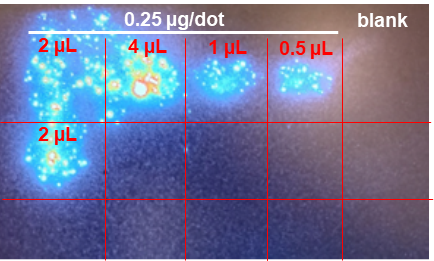


**Figure S11. Dot blotting assay testing the binding specificity of ^99m^Tc-6B11.** Autoradiography result of the dot blotting assay. CEACAM5 protein was prefixed on the membrane 1 dot per square as indicated. In the square indicated by blank, same volume of PBS was prefixed on the membrane. The volume of ^99m^Tc-6B11 incubated in each square is indicated in red.


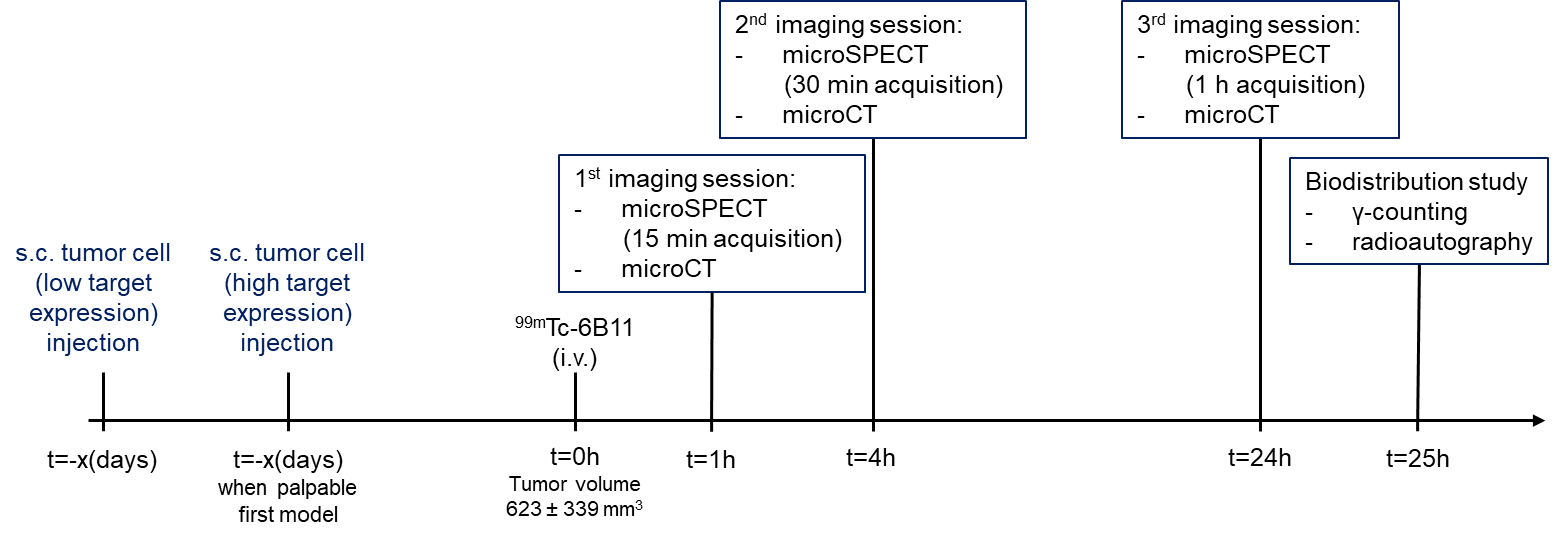


**Figure S12. Experimental set-up of the *in vivo* study.**

**Figure S13. *In vitro* viability assay.** A549-CEA5-OV and A549-CEA5-KO were treated with various concentrations of or 6F8 for 72 h and the viability of the cells was assessed by an Alamar Blue assay. Data represents the mean of n=6 repeats with SD.


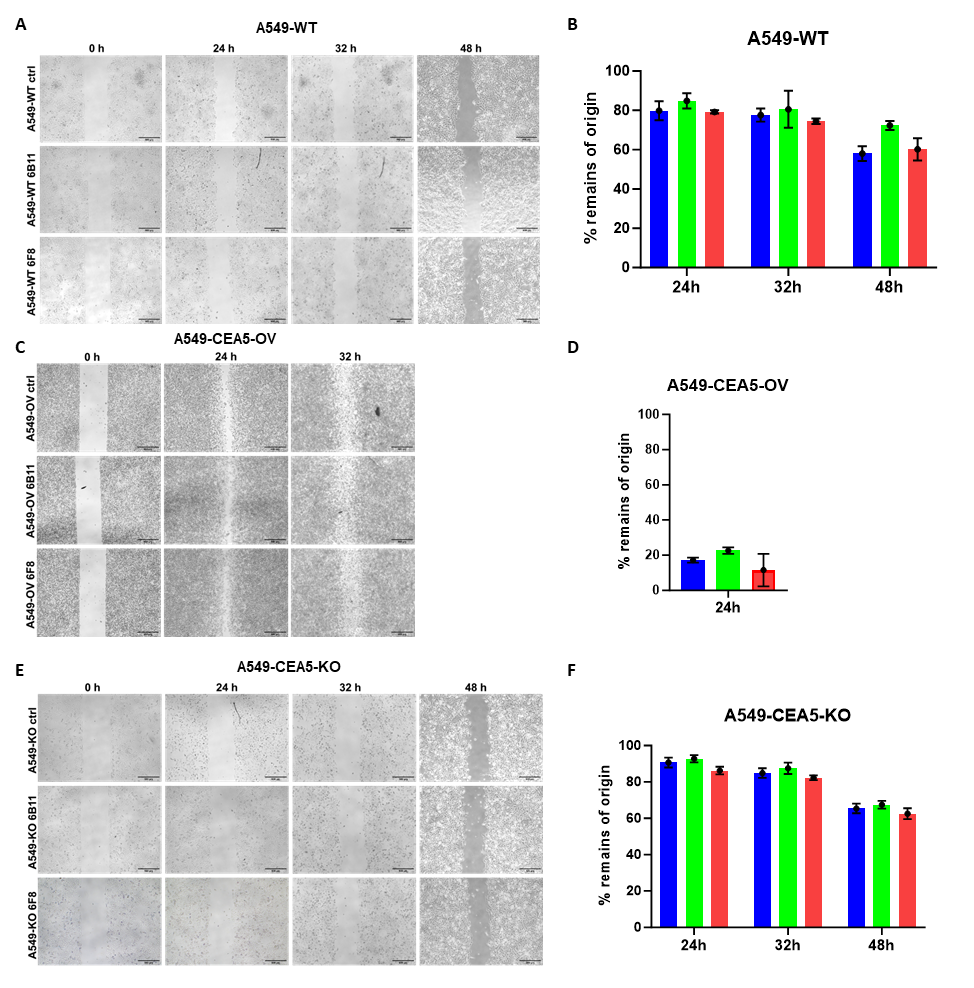


**Figure S14. Cell migration tested by wound healing assay.** Representative images of the wound healing in (A) wild type A549 (A549-WT), (C) A549 CEACAM5 overexpressing (A549-CEA5-OV) and (E) A549 CEACAM5 knockout (A549-CEA5-KO) cells treated with PBS control (ctrl), 6F8 or 6B11 VHH. Migration was quantified as percentage remaining wound area at different time points divided by the original wound area in A549-WT (B), A549-CEA5-OV (D) and A549-CEA5-KO (F) cells treated with PBS (ctrl; blue), CEACAM5 VHH 6F8 (green) or 6B11 (red). Data represent the mean of n=4 wounds with SD.

**Figure S15. *In vitro* adhesion assay.** Percentage of adhesion of A549-CEA5-OV (A) and A549-CEA5-KO (B) to fibronectin upon treatment with 6B11 or 6F8 VHH. Attached viable cells were determined by Alamar Blue assay. Data represent the mean of n=3 repeats with SD.
